# Genome-Wide Identification and Transcript Analysis Reveal Potential Roles of Oligopeptide Transporter Genes in Iron Deficiency Induced Cadmium Accumulation in Peanut

**DOI:** 10.3389/fpls.2022.894848

**Published:** 2022-05-11

**Authors:** Chaohui Wang, Xueqin Wang, Jinxiu Li, Junhua Guan, Zengjing Tan, Zheng Zhang, Gangrong Shi

**Affiliations:** College of Life Sciences, Huaibei Normal University, Huaibei, China

**Keywords:** peanut, oligopeptide transporter, cultivar, Fe deficiency, Cd accumulation

## Abstract

The oligopeptide transporter (OPT) family is a group of proton-coupled symporters that play diverse roles, including metal homeostasis. However, little is known about this family of peanuts. To reveal the potential roles of *AhOPT* genes in Fe/Cd interactions, peanut *AhOPT* genes were genome-widely identified, and the relationships between gene expression and Cd accumulation were detected in two contrasting peanut cultivars (Fenghua 1 and Silihong) under Fe-sufficient or Fe-deficient conditions. A total of 40 *AhOPT* genes were identified in peanuts, which were divided into two subfamilies (PT and YS). Most *AhOPT* genes underwent gene duplication events predominated by whole-genome duplication. Clustered members generally have similar protein structures. However, gene structural divergences occurred in most of the duplicated genes. Transcription analysis revealed that *AhOPT3.2*/*3.4* and *AhYSL3.1*/*3.2* might be responsible for Fe deficiency tolerance, while *AhOPT3.1*/*3.4, AhOPT7.1*/*7.2*, and *AhYSL1.1* be involved in Fe/Cd interactions. These genes might be regulated by transcription factors, including *ATHB-12, ATHB-6, DIVARICATA, MYB30, NAC02, DOF3.4, IDD7*, and *LUX*. Reduced expressions of *AhYSL3.1*/*3.2* and higher expressions of *AhOPT3.4* might contribute to higher Fe-deficiency tolerance in Silihong. Higher expression of *AhOPT7.3* and *AhOPT6.1* might be responsible for low Cd accumulation in Fenghua 1. Our results confirmed that *AhOPT3*/*6*/*7* and *AhYSL1*/*3* might be involved in the transport of Fe and/or Cd in peanuts and provided new clues to understanding potential mechanisms of Fe/Cd interactions.

## Introduction

Iron is an essential element for all organisms and plays crucial roles in several biological processes in plants, including chlorophyll biosynthesis, photosynthesis, respiration, nitrogen fixation, and sulfur assimilation (Marschner, 1995). Although iron (Fe) is abundant in soils, it is limited in alkaline soils (which account for approximately 30% of the world's arable land) due to insoluble Fe (III) chelates prevail (Gayomba et al., 2015). In contrast, Fe shows a high bioavailability in acidic soils that can be toxic to plants, because Fe in excess can induce the formation of reactive oxygen species through the Fenton reaction (Gayomba et al., 2015; Wu et al., 2017). Therefore, plants have evolved complex mechanisms to sense and respond to iron fluctuations in the rhizosphere, and to prevent iron deficiency or toxicity by maintaining Fe homeostasis (Gayomba et al., 2015).

Cadmium (Cd) is a non-essential heavy metal with high toxicity to almost all organisms. It is easily taken up by plants and transferred to humans/animals *via* food chains, causing serious risks to human health. Cd stress significantly reduced Fe concentrations in rice plants at low Fe levels (Shao et al., 2007). The uptake and accumulation of Cd in plants were increased by iron deficiency (Nakanishi et al., 2006; Su et al., 2013; Chen et al., 2019), while Fe supply prevents Cd uptake (Shao et al., 2007; Shi et al., 2014b). A large number of transporters have been shown to be involved in the uptake and translocation of Fe and Cd, including oligopeptide transporters (OPTs).

The OPT family is a group of proton-coupled symporters that play diverse roles in metal homeostasis, nitrogen mobilization, and sulfur distribution (Lubkowitz, 2011; Su et al., 2019). OPT proteins are predicted to contain two highly conserved motifs (NPG and KIPPR) and have 12–14 transmembrane domains (TMDs) with the N- and C-termini facing extracellular (Koh et al., 2002; Wiles et al., 2006). In *Arabidopsis*, 17 OPT members were identified and phylogenetically divided into two subfamilies: the Oligopeptide Transporter (PT) and Yellow Stripe-Like (YS) (Koh et al., 2002). YS members were found in archaea, eubacteria, fungi, and plants but not in animals, while PT genes have only been identified in plants and fungi (Lubkowitz, 2011; Su et al., 2019).

The YS subfamily members from rice and *Arabidopsis* can be divided into four groups (Curie et al., 2008), and most of them have been functionally characterized. *AtYSL1* contributes to the long-distance translocation of Fe(II)–NA *via* the xylem and delivery to the seeds (Jean et al., 2005). *AtYSL1, AtYSL2*, and *AtYSL3* are required for the efficient mobilization of Fe, Zn, and Cu from leaves to seeds (Waters et al., 2006; Chu et al., 2010). AtYSL4 and AtYSL6 are located at the internal membranes, such as chloroplast envelope, vacuole membranes, and resembling endoplasmic reticulum, mediating intracellular transport of metal-NA complexes within the cell (Conte et al., 2013; Divol et al., 2013). *OsYSL2* is a critical Fe-NA transporter required for the long-distance transport of Fe(II)-NA and Mn(II)-NA *via* the phloem (Koike et al., 2004; Ishimaru et al., 2010). OsYSL6 is an Mn-NA transporter is responsible for the detoxification of excess Mn (Sasaki et al., 2011). *OsYSL9* and *OsYSL13* are involved in Fe translocation in plants particularly from endosperm to embryo in developing seeds (Senoura et al., 2017; Zhang et al., 2018b). OsYSL15 is a Fe(III)-DMA transporter involved in Fe(III)-DMA uptake from the rhizosphere and in phloem transport of Fe in rice plants (Inoue et al., 2009; Lee et al., 2009). OsYSL16 is responsible for the allocation of Fe(III)-DMA (Kakei et al., 2012; Lee et al., 2012) and Cu(II)-NA (Zheng et al., 2012; Zhang et al., 2018a) *via* the vascular bundles. *OsYSL18* is involved in Fe(III)-DMA distribution in the reproductive organs, lamina joints, and phloem cells at the base of the leaf sheath (Aoyama et al., 2009).

Although PT subfamily genes have previously been demonstrated to transport small peptides, such as glutathione in plants (Koh et al., 2002; Bogs et al., 2003; Cagnac et al., 2004; Zhang et al., 2004; Osawa et al., 2006), they were shown to play important roles in the regulation of metal homeostasis. *AtOPT3* is predominantly expressed in the vascular tissues of leaves and reproductive organs in *Arabidopsis* and its expression was induced by Fe deficiency (Stacey et al., 2006, 2008). OPT3 loads Fe into the phloem facilitates xylem-to-phloem Fe recirculation, regulates Fe redistribution from mature to developing tissues, and mediates shoot-to-root Fe signaling (Stacey et al., 2008; Mendoza-Cózatl et al., 2014; Zhai et al., 2014). Besides, AtOPT3 is also proven to regulate the translocation and distribution of Cd in *Arabidopsis* (Mendoza-Cózatl et al., 2014; Zhai et al., 2014). *OsOPT7* expresses in root tips and vascular tissue leaves, as well as developing seeds, and was specifically upregulated by Fe-deficiency, playing an important role in Fe homeostasis under Fe-limiting conditions (Bashir et al., 2015).

Peanut (*Arachis hypogaea* L., 2*n* = 4*x* = 40) is a major oil-seed legume cash crop mainly grown in temperate and tropical regions of the world. Peanut is widely cultivated in alkaline calcareous soils and often faces iron deficiency, which seriously limits the yield and quality (Su et al., 2015). Unfortunately, the problem of iron deficiency in peanut cannot be completely solved at present, due to scarce knowledge about the molecular mechanism underlying iron uptake and transport in peanuts. More seriously, peanuts have a high capacity for accumulating Cd in both the seed and vegetative tissues (Shi et al., 2014a; Liu et al., 2017), and the uptake and accumulation of Cd in peanut plants are increased by iron deficiency (Su et al., 2013, 2014; Chen et al., 2019). However, little is known about the mechanism of Fe/Cd interaction in the process of uptake and translocation in peanuts.

Gratefully, the whole-genome sequences of the cultivated peanut (cv. Tifrunner) as well as the two wild ancestral species, *A. duranensis*, and *A. ipaënsis*, have been released (Bertioli et al., 2016, 2019). This makes it possible for the whole-genome identification of gene families in peanuts. Herein, members of the *AhOPT* family were genome-widely identified in peanuts, and their structures, functions, and evolutionary relationships were characterized. Furthermore, the expression of *AhOPT* genes in response to Fe deficiency and/or Cd exposure was investigated. Our data will provide a basis for further functional characterization of *AhOPT*s and shed new light on the possible roles of the *AhOPT* family in the uptake and translocation of Fe and Cd in plants.

## Materials and Methods

### Identification of *OPT* Genes in Peanut

To identify potential members of the OPT family in peanut genome, the protein sequences of *Arabidopsis* (17 genes) and rice (28 genes) obtained from phytozome database^1^ were used as queries for BLASTP against the peanut genome on phytozome. The candidate peanut OPT protein sequences were searched using the hmmscan tool,^2^ and the sequences containing OPT domain (PF03169) were identified as OPT proteins. Redundant OPTs were removed according to the sequence identity threshold (100%), using CD-HIT software (Li and Godzik, 2006).

### Phylogenetic Analysis

The OPT protein sequences of peanut, *Arabidopsis*, and rice were aligned by ClustalW in MEGA-X program (version 10.2.6). The aligned files were used to construct a phylogenetic tree using the neighbor-joining (NJ) method based on the Poisson model with 1,000 bootstrap replicates. The constructed data were used for plotting the evolutionary tree on an online software iTOL.^3^

### Physicochemical and Structural Characteristics of AhOPT Proteins

Physiochemical properties of AhOPT proteins were estimated using the ProtParam tool^4^ (Duvaud et al., 2021). TMD numbers were predicted using TOPCONS^5^ (Tsirigos et al., 2015). Subcellular localization of AhOPT proteins was predicted with Plant-mPLoc^6^ (Chou and Shen, 2010). The conserved motifs and domains in AhOPT sequences were examined using the MEME version 5.3.3 (v. 5.3.3)^7^ (Bailey et al., 2006) and Pfam tool^8^ (Mistry et al., 2020), respectively.

### Exon-Intron Structure, Gene Duplication, *K*a/*K*s, and MicroRNA Target Sites of *AhOPTs*

The exon-intron structure of all *AhOPT* genes was determined using GSDS (v. 2)^9^ (Hu et al., 2015). Gene collinearity and Ka/Ks (ratios of the number of non-synonymous substitutions per non-synonymous site to the number of synonymous substitutions per synonymous site) were analyzed by One Step MCScanX and simple *K*a/*K*s calculator (NJ) of TBtools software, respectively (Chen et al., 2020). Diagrams of exon-intron organization and gene duplication event were drawn using TBtools Software (Chen et al., 2020). To better explain the patterns of macroevolution, The Ks value was used to calculate the divergence times of the duplication event (T = *K*s/2λ), the neutral substitution rate (λ) is estimated to be 8.12 × 10^−9^ for peanut (Bertioli et al., 2016). MicroRNA target sites were analyzed by psRNATarget (Dai et al., 2018).

### Tissue-Specific Expression Profiles of *AhOPT* Genes in Peanut

Tissue-specific expression profiles of *AhOPT* genes were identified using RNA-seq data of cv. Tifrunner obtained from PeanutBase^10^ (Clevenger et al., 2016). Read counts were transformed to fragments per kilobase of exon per million aligned fragments (FPKM), and the heatmap diagram was constructed with lg^(FPKM+1)^ using TBtools (Chen et al., 2020).

### Plant Growth and Treatment

Two contrasting peanut cultivars, Fenghua 1 (Fe deficiency sensitive/Cd tolerant cultivar) and Silihong (Fe deficiency tolerant but Cd sensitive cultivar), were used for determining Cd accumulation in peanut plants (Liu et al., 2017; Tian et al., 2019). The seeds were surface sterilized with 5% sodium hypochlorite (1 min), soaked in distilled water for 24 h, and then sown in sand for germination. Three-d-old uniform seedlings were transferred to polyethylene pots and cultured as previously reported (Su et al., 2014). The 7-d-old seedlings were treated with 0 or 2 μM CdCl_2_ in hydroponic cultures, under Fe-sufficient (50 μM Fe-EDTA) or Fe-deficient (0 μM Fe-EDTA) conditions, respectively. The experiment was arranged in a randomized complete design with triplications (pots) for each treatment. Each replication includes three seedlings. Plants were cultivated in a growth chamber under 14-h photoperiod (average irradiance of 632 μmol m^−2^ s^−1^), day/night temperature of 27.4 ± 2.2/23.1 ± 1.6°C and day/night relative humidity of 68 ± 6/75 ± 4%. During the growing period, pots were randomly arranged and moved daily for minimizing position effects. After 14 days of treatment, plants were harvested and fresh root tissues were sampled for RT-qPCR analysis.

### Cadmium Determination

The harvested plants were separated into roots and shoots, and then, the roots were rinsed with 20 mM Na_2_EDTA for 15 min to remove surface-bound metal ions. After oven-drying, the roots and shoots were weighed and ground into powder. Samples of the roots (0.1 g) and shoots (0.5 g) were digested with HNO_3_-HClO_4_ (3:1, v/v) as the method described by Su et al. (2014). Cd concentrations were determined by flame atomic absorbance spectrometry (WFX-110, Beijing Rayleigh Analytical Instrument Company, China). The root-to-shoot translocation of Cd was indicated as the percentage of Cd in shoots, which were calculated as the following equation:


$$\begin{array}{l} \%\text{ of Cd in shoots }=\\ 100\times \frac{\text{shoot DW }\times \text{ Cd con}.\text{ in shoots}}{\left(\text{shoot DW }\times \text{ Cd con}.\text{ in shoots }+\text{ root DW }\times \text{ Cd con}.\text{ in roots}\right)} \end{array}$$

### Transcriptional Responses of *AhOPT* Genes to Fe Deficiency and Cd Exposure

Expression profiles of *AhOPT* genes in the roots of Fenghua 1 and Silihong in different Fe/Cd treatments were analyzed using RNA-seq data, which have been published previously (Cao et al., 2019; Chen et al., 2019). The heatmap diagram was constructed with lg^(FPKM+1)^ using TBtools (Chen et al., 2020). Differentially expressed genes (DEGs) were detected using the DESeq2 R package (v. 1.16.1). Genes with the fold change (FC) least 2 times higher or lower (|log2FC| ≥ 1) and *p*-values adjusted by the Benjamini-Hochberg method (*P*_adj_) < 0.05 were defined as DEGs.

### Prediction and Screening of Transcription Factors of *AhOPT* Genes

Transcription factors (TFs) of *AhOPT* genes were identified using the PlantRegMap database^11^ (Tian et al., 2019). Since the database does not contain the information on cultivated peanuts, we firstly obtained the most similar ortholog of each *AhOPT* gene from the genomes of *A. duranensis* and *A. ipaënsis* by BLASTP on peanutbase database.^12^ The obtained gene IDs were used for predicting TFs on the PlantRegMap database and subsequently, their sequences were used as queries for BLASTP against the peanut genome. The most homologous ortholog in each query is considered to be the possible TF of *AhOPT* genes.

Pearson correlation coefficient values were used for evaluating the co-expression correlation between *AhOPT* genes and their TFs, based on FPKM values of RNA-seq data in different Fe/Cd treatments. The pairs with *P* < 0.05 are determined as existing co-expression correlation, while *P* < 0.01 was determined as an existing strong co-expression correlation. The network of co-expressed genes was constructed using Cytoscape (v. 3.7.1).

### RT-qPCR Analysis

The expression levels of ten *AhOPT* genes that were differently expressed in Fe and/or Cd treatments, as well as two TFs, were detected using RT-qPCR as the method described previously (Cao et al., 2019), with *Ah60S* as the endogenous reference gene. The primers are listed in Supplementary Table 1. Three technical replications were carried out for each treatment. The relative gene expression was calculated using the 2^−ΔΔCT^ method.

### Statistical Analysis

Data were subjected to a one-way analysis of variance, and significant differences among means were determined by Duncan's Multiple Range Test at a probability level of 5%. Pearson's correlation analysis was performed to examine the relationships between the gene expression and Cd accumulation in peanut roots. All data analysis was carried out using the IBM SPSS Statistics version 22 (IBM, New York, USA).

## Results

### Summary of The *AhOPT* Gene Family in Peanut

A total of 40 putative *AhOPT* genes were identified in peanuts, including four *AhOPT3*, eight *AhOPT4*, six *AhOPT5*, two *AhOPT6*, six *AhOPT7*, two *AhYSL1*, two *AhYSL3*, two *AhYSL6*, and eight *AhYSL7* (Table 1). The length of *AhOPT* genes varies greatly, ranging from 1,180 bp (*AhOPT3.3*) to 12,616 bp (*AhOPT7.6*), with CDS lengths from 396 bp (*AhOPT3.1*) to 4,197 bp (*AhYSL3.2*). The amino acid number of AhOPT proteins varied from 132 (AhOPT3.1) to 1,399 (AhYSL3.2), and the corresponding molecular weight varied from 14.9 kDa (AhOPT3.1) to 154.3 kDa (AhYSL3.2). The instability index for 90% AhOPT proteins was lower than 40, indicating high stability *in vitro*. All proteins of the AhOPT family showed a high aliphatic index (91.1–106.9), implying these proteins might be stable over a wide temperature range. The GRAVY of all AhOPT proteins are higher than 0 (ranged from 0.19 to 0.56), suggesting AhOPTs are hydrophobic proteins. Most of AhOPT proteins (90%) are basic proteins (pI > 7), and only four AhOPTs (AhYSL6.1, AhYSL6.2, AhOPT7.3, and AhOPT7.6) are acidic proteins (pI < 7) (Table 1). The number of TMDs widely varied among AhOPT proteins (ranging from 1 to 30), and most AhOPTs contained 11–17 TMDs (Table 1). All AhOPT proteins were predicted to be localized in plasma membranes (Table 1).

**Table 1:** Molecular characterization of *AhOPT* genes identified in peanut.

**Gene name**	**Gene ID**	**Gene length (bp)**	**CDS (bp)**	**MW (kDa)**	**aa**	**Instability**	**Aliphaticindex**	**GRAVY**	**pI**	**No. of TMD**	**Location**
*AhOPT3.1*	arahy.F397ET	1,238	396	132	14.9	29.2	96.7	0.41	9.06	1	PM
*AhOPT3.2*	arahy.XKSD0A	4,711	2,229	743	83.5	31.1	102.5	0.42	8.95	15	PM
*AhOPT3.3*	arahy.6RC7K9	1,180	549	183	20.6	43.9	95.4	0.50	8.44	3	PM
*AhOPT3.4*	arahy.WI2A41	4,704	2,229	743	83.5	30.9	102.5	0.42	9.00	15	PM
*AhOPT4.1*	arahy.7XWF6F	3,727	2,232	744	83.6	32.5	99.5	0.43	9.11	14	PM
*AhOPT4.2*	arahy.4S3D7T	3,567	2,232	744	83.4	31.2	99.6	0.44	8.97	14	PM
*AhOPT4.3*	arahy.6W1ZCJ	2,191	1,542	514	58.0	24.2	104.7	0.54	9.16	11	PM
*AhOPT4.4*	arahy.BCI2ZL	6,214	2,361	787	88.5	29.2	94.8	0.34	9.21	14	PM
*AhOPT4.5*	arahy.VRJ6U0	3,831	2,232	744	83.5	32.2	98.8	0.42	9.02	14	PM
*AhOPT4.6*	arahy.05TCFP	4,027	2,232	744	83.4	31.3	99.2	0.43	8.92	14	PM
*AhOPT4.7*	arahy.438BUP	4,388	1,590	530	60.8	36.0	91.1	0.21	8.46	8	PM
*AhOPT4.8*	arahy.QC3XVA	6,480	2,328	776	87.0	29.7	94.2	0.35	9.09	14	PM
*AhOPT5.1*	arahy.UU5TC6	10,264	1,950	650	72.7	29.6	103.3	0.43	9.35	11	PM
*AhOPT5.2*	arahy.6WW0WD	2,999	1,458	486	54.5	31.6	101.1	0.32	8.98	9	PM
*AhOPT5.3*	arahy.BL3QR7	5,166	1,971	657	73.7	35.2	103.7	0.36	8.71	9	PM
*AhOPT5.4*	arahy.NEQX61	7,893	2,247	749	83.8	32.6	104.0	0.38	9.12	16	PM
*AhOPT5.5*	arahy.QU3QAE	4,557	2,208	736	82.2	31.7	103.4	0.42	9.10	16	PM
*AhOPT5.6*	arahy.ZV39IJ	8,348	1,980	660	74.0	28.8	100.9	0.40	8.79	14	PM
*AhOPT6.1*	arahy.K3DJI3	4,191	1,908	636	71.7	31.4	93.2	0.42	9.49	13	PM
*AhOPT6.2*	arahy.Y086TD	4,328	2,088	696	78.6	31.2	94.8	0.43	9.21	13	PM
*AhOPT7.1*	arahy.XJAC58	5,625	2,244	748	84.3	42.6	102.8	0.41	7.28	14	PM
*AhOPT7.2*	arahy.6K1PAL	10,476	2,700	900	100.5	41.2	93.2	0.19	8.22	15	PM
*AhOPT7.3*	arahy.XXTB4R	12,211	2,220	740	83.5	37.9	100.3	0.43	6.88	14	PM
*AhOPT7.4*	arahy.0Z487T	9,776	2,700	900	100.6	42.2	93.7	0.19	8.06	14	PM
*AhOPT7.5*	arahy.YIY9X1	4,945	2,337	779	87.9	39.5	99.8	0.39	7.86	14	PM
*AhOPT7.6*	arahy.KDG5NW	12,616	1,899	633	71.3	33.9	99.8	0.46	5.83	11	PM
*AhYSL1.1*	arahy.WL6ZXR	4,492	2,037	679	75.0	31.1	101.1	0.48	9.12	17	PM
*AhYSL1.2*	arahy.T6ZY4C	4,511	2,037	679	75.0	31.5	100.2	0.48	9.07	17	PM
*AhYSL3.1*	arahy.HMP3A6	9,096	4,092	1,364	150.0	33.0	103.4	0.45	8.73	30	PM
*AhYSL3.2*	arahy.53J40D	9,896	4,197	1,399	154.3	34.7	105.8	0.42	8.67	26	PM
*AhYSL6.1*	arahy.WJ50T5	7,840	2,013	671	73.3	29.5	100.1	0.53	5.96	16	PM
*AhYSL6.2*	arahy.L877Z8	7,678	2,106	702	76.9	30.4	102.6	0.56	5.90	14	PM
*AhYSL7.1*	arahy.B192XI	5,640	2,088	696	75.7	31.3	95.0	0.49	8.59	16	PM
*AhYSL7.2*	arahy.06T2K2	5,045	2,112	704	77.5	32.0	98.9	0.39	9.12	16	PM
*AhYSL7.3*	arahy.Z73NZ6	2,627	975	325	35.2	27.3	106.9	0.55	9.06	7	PM
*AhYSL7.4*	arahy.ZW21UW	3,940	2,067	689	76.4	34.1	94.4	0.35	8.95	16	PM
*AhYSL7.5*	arahy.EJV4EX	5,610	2,109	703	76.6	30.9	95.1	0.46	8.68	16	PM
*AhYSL7.6*	arahy.6K67PT	3,176	1,212	404	44.6	30.4	98.3	0.53	8.42	7	PM
*AhYSL7.7*	arahy.4749WV	4,409	2,226	742	82.5	32.0	92.6	0.24	9.17	14	PM
*AhYSL7.8*	arahy.09HEKB	6,385	1,740	580	64.1	28.8	97.7	0.33	9.15	13	PM

*MW, Molecular weight; aa, Amino acid number; GRAVY, Grand average of hydropathicity; pI, Isoelectric points; TMD, Transmembrane domain; PM, Plasma membrane*.

### Phylogenetic Analysis of OPT Proteins

**Figure 1:**
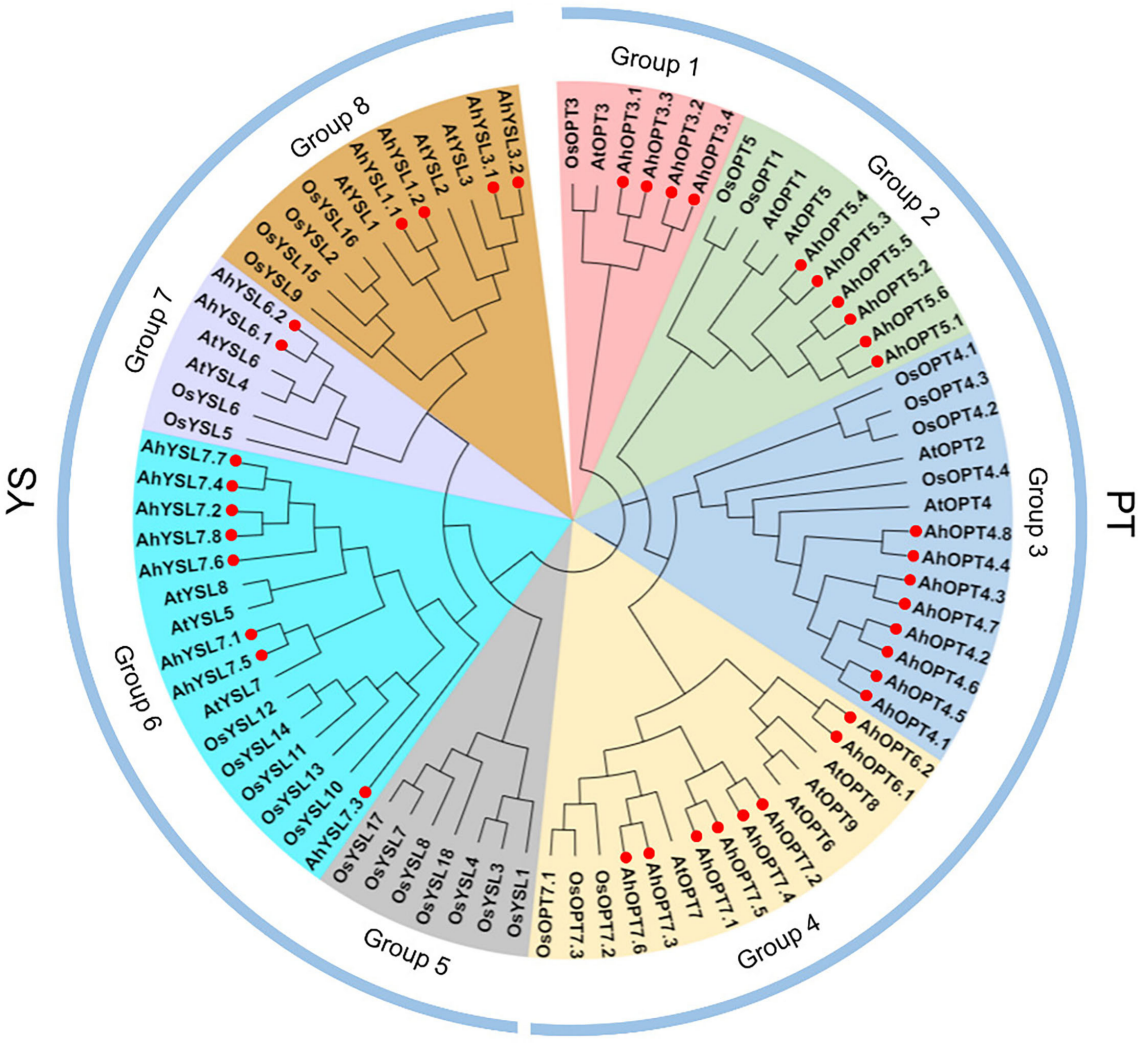
Phylogenetic relationships of oligopeptide transporter (OPT) proteins from peanut, *Arabidopsis*, and rice. Red solid circles represent the 40 AhOPT proteins of peanut.

The phylogenetic relationship of 85 OPTs from peanut, *Arabidopsis*, and rice was analyzed with the NJ method. OPT proteins were divided into two subfamilies (PT and YS) (Figure 1). The 26 AhOPT proteins assigned to the PT subfamily were further classified into four groups: group 1 (AhOPT3.1/3.2/3.3/3.4), group 2 (AhOPT5.1/5.2/5.3/5.4/5.5/5.6), group 3 (AhOPT4.1/4.2/4.3/4.4/4.5/4.6/4.7/4.8), and group 4 (AhOPT6.1/6.2 and AhOPT7.1/7.2/7.3/7.4/7.5/7.6). The remaining 14 members of peanut were clustered into three groups of the YS subfamily, including group 6 (AhYSL7.1/7.2/7.3/7.4/7.5/7.6/7.7/7.8), group 7 (AhYSL6.1/6.2), and group 8 (AhYSL1.1/1.2 and AhYSL3.1/3.2) (Figure 1). No AhYSL member was included in group 5, which was occupied by seven YSLs from rice.

### Conserved Motifs, Domain Architectures, and Models of AhOPT Proteins

**Figure 2:**
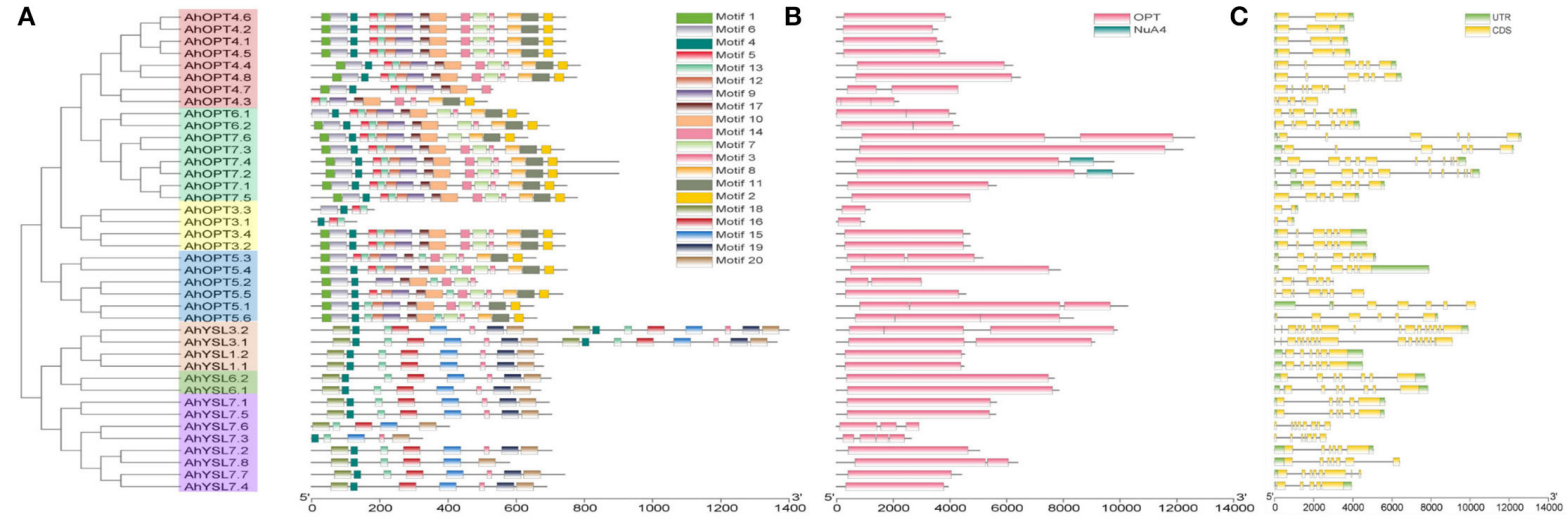
The conserved motifs **(A)**, domains **(B)**, and of AhOPT proteins as well as the exon-intron organization **(C)** of *AhOPT* genes in peanut.

The AhOPT proteins at least contained 20 conserved motifs, and most of them were annotated to be the OPT domains according to the InterProScan and Pfam tools (Figure 2A; Supplementary Table 2). Almost all AhOPT proteins shared motif 3 (contained KIPPR), 4 (contained NPG), and 13, which were annotated to be OPT domains. The two subfamilies differed from each other in the pattern of conserved motifs. Most members of the PT subfamily contained 10–15 motifs, while the YS subfamily generally contained eight motifs. We found 12 motifs (Motif 1, 2, 5, 6, 7, 8, 9, 10, 11, 12, 14, and 7) unique to the PT subfamily and five motifs (Motif 15, 16, 18, 19, and 20) unique to the YS subfamily, respectively (Figure 2A). The composition of conserved motifs was similar within the phylogenetic subfamily or group. However, several AhOPT proteins, such as AhOPT4.3/4.7, AhOPT3.1/3.3, AhOPT5.2, and AhYSL7.3/7.6, contained distinctive motifs (Figure 2A).

All AhOPT proteins contained the typical domain, OPT (Figure 2B). However, the number of conserved domain varied among AhOPT proteins. While most proteins contained one OPT domain, AhOPT4.7, AhOPT5.2, AhOPT6.1/6.2, AhOPT7.6, AhYSL3.1, AhYSL7.8 contained two OPT domains, AhOPT4.3, AhOPT5.1/5.3/5.6, AhYSL3.2, and AhYSL7.6 contained three OPT domains, and AhYSL7.3 contained four OPT domains. An additional domain, NuA4 (PF09340, Nucleosome acetyltransferase of histone H4), was identified in AhOPT7.2 and AhOPT7.4 (Figure 2B).

### Structure and Duplication of *AhOPT* Genes

To gain insight into the evolution of the OPT family in peanuts, exon-intron organizations were examined. As presented in Figure 2C, *AhOPT* genes showed large divergences in exon-intron organizations. The majority of the *AhOPT* genes contain six or seven exons, whereas *AhOPT3.1*/*3.3* has only two exons (one intron), and *AhYSL3.1*/*3.2* has 17 exons (16 introns). Although several pairs of *AhOPT* genes, such as *AhOPT4.1*/*4.5* (3 exons and 2 introns), *AhOPT4.2*/*4.6* (3 exons and 2 introns), *AhOPT3.2*/*3.4* (6 exons and 5 introns), *AhYSL1.1*/*1.2* (6 exons and 6 introns), and *AhYSL7.1*/*7.5* (6 exons and 5 introns), possess similar exon-intron structure, various exon-intron devergences were identified in the remaining gene pairs.

**Figure 3:**
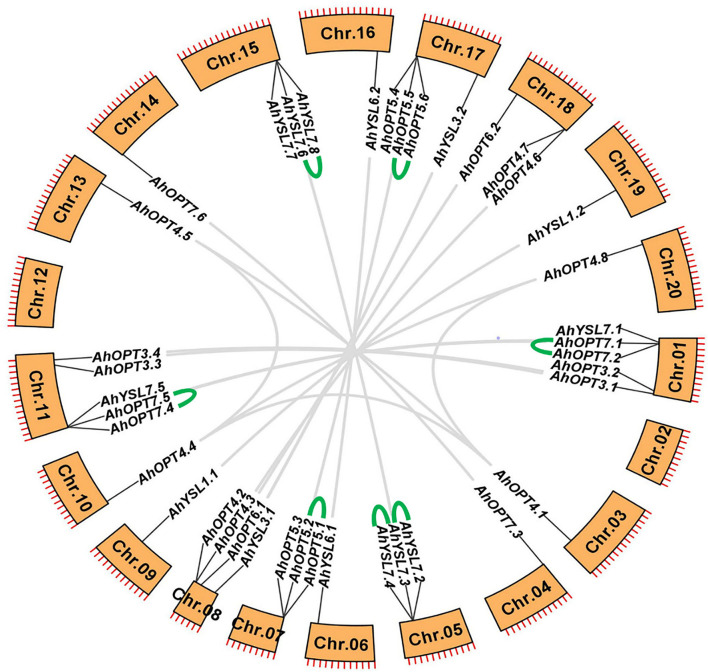
Chromosomal locations and gene duplication of *AhOPT* genes. The gene pairs derived from whole-genome duplication (or segmental duplication) and tandem duplication are linked by gray and green lines, respectively.

The 40 *AhOPT* genes were distributed unevenly in 18 chromosomes, with chromosomes 02 and 12 being devoid of *AhOPT* genes. Each of the two subgenomes (subgenome A, chromosome 01–10; subgenome B, chromosome 11–20) has 20 *AhOPT* genes (Figure 3). Chromosomes 01 and 11 contained the largest number of *AhOPT* genes (five genes per chromosome), followed by chromosomes 08 and 17 (four genes per chromosome), and the least genes were presented on chromosomes 03, 04, 09, 10, 13, 14, 19, and 20 (one gene per chromosome).

Collinearity analysis revealed that the *AhOPT* family experienced very complex gene duplication, resulting in a large number of multicopy genes (Figure 3). Sixteen *AhOPT* genes of the subgenome A were crossly collineared with corresponding genes of the subgenome B, forming 16 gene pairs. These collinear blocks could be considered as whole-genome duplications (WGDs). *AhOPT4.1*/*4.4* might result from segmental duplication because they are in different chromosomes of the same subgenome. Additionally, tandem duplication events were also occurred in seven gene pairs, including *AhYSL7.2/7.3, AhYSL7.3/7.4, AhYSL7.6/7.8, AhOPT7.1/7.2, AhOPT7.4/7.5, AhOPT5.1/5.2*, and *AhOPT5.5/5.6* (Figure 3). The *K*a/*K*s ratios of all gene duplication pairs were <1 (Table 2), indicating that *AhOPT* genes evolved under purifying selection (Hurst, 2002).

**Table 2:** *Ka*/*Ks* analysis of all gene duplication pairs for *AhOPT* genes.

**Duplicated pair**	**Duplicate type**	***Ka***	***Ks***	***Ka*/*Ks***	**Positive selection**	**Divergence time (Mya)**
*AhOPT4.1*/*4.4*	Segmental	0.115	1.091	0.106	No	67.17
*AhOPT4.1*/*4.8*	Segmental	0.121	1.042	0.116	No	64.15
*AhOPT4.4*/*4.5*	Segmental	0.117	1.058	0.110	No	65.17
*AhOPT5.1*/*5.2*	Tandem	0.121	0.122	0.996	No	7.50
*AhOPT5.5*/*5.6*	Tandem	0.098	0.150	0.651	No	9.26
*AhOPT7.1*/*7.2*	Tandem	0.120	0.852	0.141	No	52.45
*AhOPT7.4*/*7.5*	Tandem	0.122	0.862	0.141	No	53.09
*AhYSL7.2*/*7.3*	Tandem	0.118	0.521	0.226	No	32.07
*AhYSL7.3*/*7.4*	Tandem	0.187	0.559	0.334	No	34.43
*AhYSL7.6*/*7.8*	Tandem	0.150	0.535	0.280	No	32.91
*AhOPT3.1*/*3.3*	Whole-genome	0.012	0.051	0.230	No	3.11
*AhOPT3.2*/*3.4*	Whole-genome	0.001	0.033	0.036	No	2.03
*AhOPT4.1*/*4.5*	Whole-genome	0.007	0.031	0.210	No	1.91
*AhOPT4.2*/*4.6*	Whole-genome	0.005	0.053	0.100	No	3.27
*AhOPT4.3*/*4.7*	Whole-genome	0.035	0.116	0.299	No	7.13
*AhOPT4.4*/*4.8*	Whole-genome	0.006	0.038	0.150	No	2.31
*AhOPT5.1*/*5.4*	Whole-genome	0.148	0.710	0.209	No	43.69
*AhOPT6.1*/*6.2*	Whole-genome	0.012	0.049	0.251	No	3.04
*AhOPT7.1*/*7.4*	Whole-genome	0.113	0.837	0.135	No	51.56
*AhOPT7.3*/*7.6*	Whole-genome	0.011	0.052	0.215	No	3.22
*AhYSL1.1*/*1.2*	Whole-genome	0.005	0.028	0.182	No	1.74
*AhYSL3.1*/*3.2*	Whole-genome	0.014	0.040	0.346	No	2.47
*AhYSL6.1*/*6.2*	Whole-genome	0.005	0.032	0.144	No	1.97
*AhYSL7.1*/*7.5*	Whole-genome	0.019	0.066	0.295	No	4.06
*AhYSL7.2*/*7.7*	Whole-genome	0.083	0.472	0.175	No	29.08
*AhYSL7.3*/*7.6*	Whole-genome	0.183	0.337	0.542	No	20.74

### The MicroRNA Target Sites of *AhOPT* Genes

A total of six miRNAs were identified, including *ahy-miR156a, ahy-miR156c, ahy-miR159, ahy-miR167-3p, ahy-miR3521*, and *ahy-miR408-5p* (Table 3). The UPE varied from 6.92 (*ahy-miR156a*/*AhOPT3.3*) to 21.55 (*ahy-miR3521*/*AhOPT7.1*). *AhOPT3.2/3.3/3.4* was predicted to be target genes of *ahy-miR156a*. Among them, *AhOPT3.2* was potential targets of *ahy-miR156c* and *ahy-miR3521*, and *AhOPT3.4* was the target of ahy-miR156c. *Ahy-miR159* possibly targets to *AhYSL3.1* and *AhYSL3.2*, and *AhYSL7.4* and *AhYSL7.7* might be the potential targets of *ahy-miR167-3p*. *AhOPT7.1* and *AhOPT7.5* were potential target genes of *ahy-miR3521*. Potential target genes of *ahy-miR408-5p* were *AhYSL7.2* and *AhYSL7.8*. All miRNAs were predicted to down-regulate the expression of corresponding target genes by cleavage of mRNA.

**Table 3:** Prediction of miRNAs for *AhOPT* transcripts.

**miRNA**	**Target**	**Expectation**	**UPE**	**miRNA aligned**	**Target aligned**	**Inhibition**
ahy-miR156a	*AhOPT3.2*	4	16.79	UGACAGAAGAG	AUGCUCUGUCU	Cleavage
				AGAGAGCAC	UUCUUGUCA	
ahy-miR156a	*AhOPT3.3*	3.5	6.92	UGACAGAAGAG	UUGCUCUCUUU	Cleavage
				AGAGAGCAC	CUUUUUUCC	
ahy-miR156a	*AhOPT3.4*	4	16.69	UGACAGAAGAG	AUGCUCUGUCU	Cleavage
				AGAGAGCAC	UUCUUGUCA	
ahy-miR156c	*AhOPT3.2*	4	16.41	UUGACAGAAGA	AUGCUCUGUCU	Cleavage
				GAGAGAGCAC	UUCUUGUCAG	
ahy-miR156c	*AhOPT3.4*	4	16.40	UUGACAGAAGA	AUGCUCUGUCU	Cleavage
				GAGAGAGCAC	UUCUUGUCAG	
ahy-miR159	*AhYSL3.1*	3.5	17.48	UUUGGAUUGAA	AUGAGCUUCUU	Cleavage
				GGGAGCUCUA	UUCAUCCAAG	
ahy-miR159	*AhYSL3.1*	4	17.11	UUUGGAUUGAA	AUGAGCUUCUU	Cleavage
				GGGAGCUCUA	UACAUCCAAA	
ahy-miR159	*AhYSL3.2*	3.5	17.48	UUUGGAUUGAA	AUGAGCUUCUU	Cleavage
				GGGAGCUCUA	UUCAUCCAAG	
ahy-miR167-3p	*AhYSL7.4*	4.5	15.90	AGAUCAUGUGG	GAUCAGAUUCC	Cleavage
				CAGUUUCACC	CACAUGGUUU	
ahy-miR167-3p	*AhYSL7.7*	4.5	16.21	AGAUCAUGUGG	GAUCAGAUUCC	Cleavage
				CAGUUUCACC	CACAUGGUUU	
ahy-miR3521	*AhOPT3.2*	4	19.74	UGGUGAGUCGU	AUGCAUGUAUA	Cleavage
				AUACAUACUG	CGGCUCAGCU	
ahy-miR3521	*AhOPT7.1*	4.5	21.55	UGGUGAGUCGU	GAGUGUUUAUG	Cleavage
				AUACAUACUG	CGAUUCAUUU	
ahy-miR3521	*AhOPT7.5*	4.5	21.38	UGGUGAGUCGU	GAGUGUUUAUG	Cleavage
				AUACAUACUG	CGAUUCAUUU	
ahy-miR408-5p	*AhYSL7.2*	4.5	15.29	CUGGGAACAGG	GAAUGUUCUCC	Cleavage
				CAGAGCAUGA	CUGUUGCUGG	
ahy-miR408-5p	*AhYSL7.8*	4.5	15.29	CUGGGAACAGG	GAAUGUUCUCC	Cleavage
				CAGAGCAUGA	CUGUUGCUGG	

*This article was submitted to Plant Nutrition, a specialty of Frontiers in Plant Science*.

### Expression Profiles of *AhOPT* Genes in Different Tissues of Peanut

**Figure 4:**
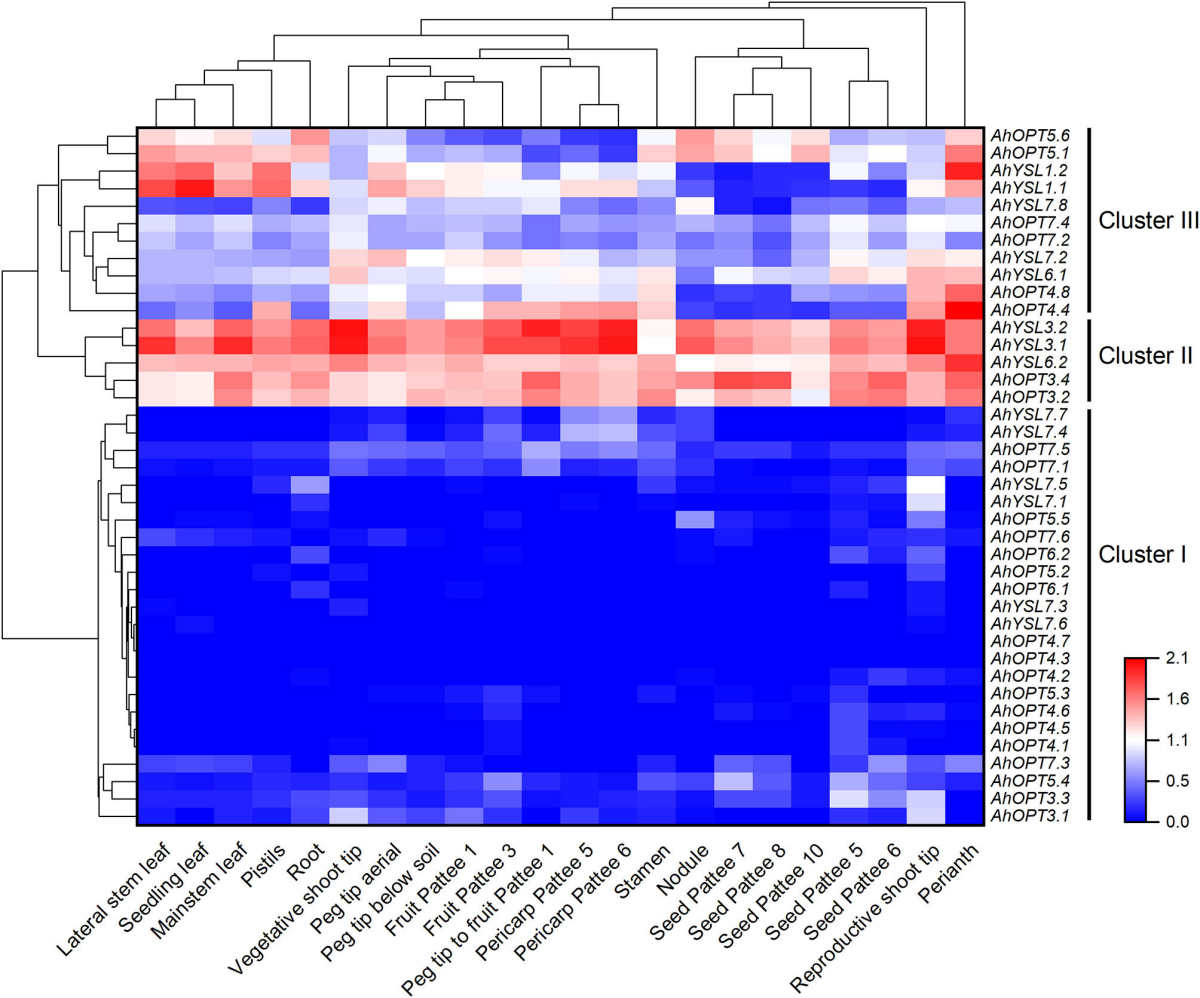
The expression profiles of *AhOPT* genes. Gene expression is expressed in lg^(FPKM+1)^. Pattee 1, 3, 5, 6, 7, 8, and 10 represent different developmental stages of peanut pods according to Pattee's classification.

The RNA-seq data showed that all *AhOPT* genes expressed in peanut tissues except *AhOP4.7*, which did not express in any of the 22 tissues (Supplementary Table 3). To better understand the expression profiles, a hierarchical cluster analysis was performed. As presenting in Figure 4, 40 *AhOPT* genes were divided into three clusters. Cluster I includes 24 genes with low expression levels, and most of them belong to the PT subfamily and group 6 of the YL subfamily. Among them, several genes, such as *AhYSL7.1*/*7.5* and *AhOPT3.1*/*3.3*, are mainly transcribed in shoot tips. *AhOPT3.3* and *AhOPT5.4* were also observed to express in fruit and seed. Cluster II consists of five *AhOPT* genes with high expression levels in all tissues, including *AhYSL6.2, AhYSL3.1/3.2*, and *AhOPT3.2*/*3.4*. Cluster III is composed of the remaining eleven genes with an intermediate level of expression, and most of them showed tissue-specific gene expression. For instance, *AhYSL6.1, AhYSL7.2*, and *AhOPT4.4*/*4.8* are preferentially transcribed in shoot tips and reproductive tissues. Additionally, *AhYSL1.1*/*1.2* and *AhOPT5.1*/*5.6* are highly and preferentially expressed in roots, leaves, and flower tissues including perianth, pistils, and stamen.

### Influence of Fe Deficiency on Cadmium Accumulation in Two Peanut Cultivars

**Figure 5:**
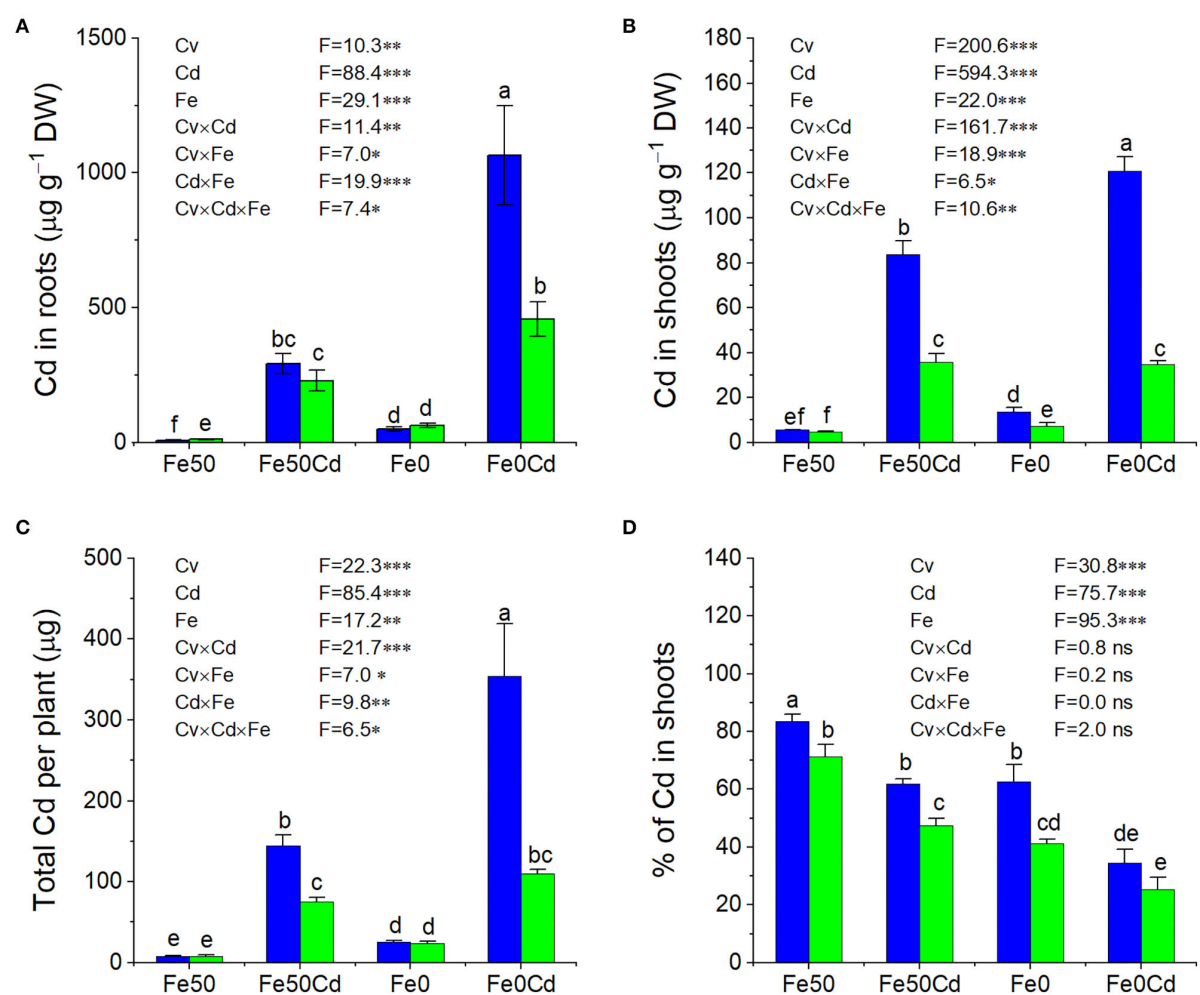
The concentration of Cd in roots **(A)**, shoots **(B)**, total Cd uptake **(C)**, and translocation of Cd **(D)** in Fe-sufficient (Fe50) or Fe-deficient (Fe0) plants of Silihong (blue columns) and Fenghua 1 (green columns) exposed to 0 or 2 μM CdCl_2_ for 14 days. Data (means ± SE, *n* = 3) shared the same letter(s) above the error bars are not significantly different at the 0.05 level by Duncan's multiple range test.

The two peanut cultivars differed from each other in Cd accumulation, which was significantly influenced by Fe deficiency (Figure 5). Generally, Silihong showed higher Cd concentrations in roots and shoots, and higher total amounts of Cd in plants than Fenghua 1 (Figures 5A–C). There are significant interactive effects between cultivar and Fe supply on Cd concentrations in roots and shoots as well as total amounts of Cd in plants (Figures 5A–C), indicating that Fe deficiency enhanced Cd uptake and accumulation in peanut plants in a cultivar-dependent manner. By contrast, Fe deficiency-induced increase of Cd accumulation was more pronounced in Silihong than in Fenghua 1. For instance, Cd concentrations in shoots and total amounts of Cd in plants were significantly enhanced by Fe deficiency in Silihong, whereas in Fenghua 1, they remained unaffected. The percentage of Cd in shoots, which as an indicator for the translocation of Cd from roots to shoots, also showed significant cultivar differences (Figure 5D). Higher percentages of Cd in shoots throughout treatments indicate that Silihong had a higher capacity for root-to-shoot Cd translocation than Fenghua 1. Fe deficiency considerably reduced the percentage of Cd in shoots regardless of cultivars.

### Transcriptional Responses of *AhOPTs* to Fe-Deficiency and Cd Exposure

**Figure 6:**
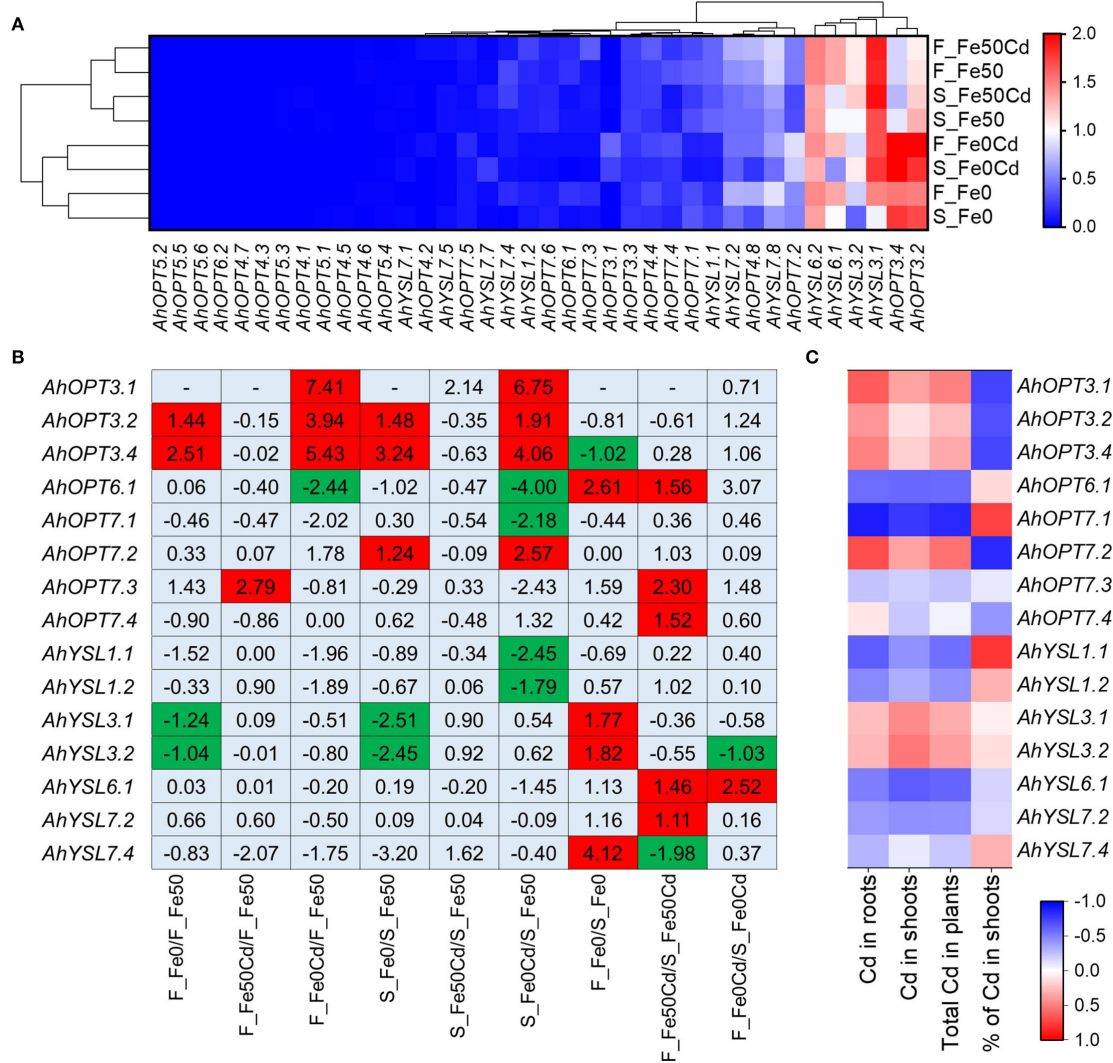
Expression profiles and DGEs of *AhOPT* genes. **(A)** Heatmap and hierarchical clustering analysis of *AhOPT* genes in Fe-sufficient (Fe50) or Fe-deficient (Fe0) roots of Silihong (S) and Fenghua 1 (F) exposed to 0 or 2 μM Cd for 14 days. **(B)** Differentially expressed *AhOPT* genes. **(C)** Correlation between the expression of differentially expressed *AhOPT* genes and Cd accumulation in peanut plants.

Cultivar difference in the transcriptional responses of *AhOPTs* to Fe deficiency and Cd exposure was investigated by using transcription data. The heat map analysis revealed two distinct clusters, representing high and low expression levels, respectively (Figure 6A). In agreement with tissue-specific expression profiling (Figure 4), *AhYSL6.1/6.2, AhYSL3.1/3.2*, and *AhOPT3.2*/*3.4* was found to show constitutive expression in all treatments. Eight treatments were clustered into two groups: Fe-sufficient and Fe-deficient groups, indicating that transcriptional profiling of *AhOPT*s was significantly influenced by Fe deficiency for both cultivars (Figure 6A). Cd did not affect transcriptional profiling of *AhOPT*s under Fe-sufficient conditions. However, under Fe-deficient conditions, Cd considerably altered the expression of *AhOPT* genes.

A total of fifteen DEGs were identified in the *AhOPT* family (Figure 6B). Among them, *AhYSL3.1*/*3.2, AhOPT3.2*/*3.4*, and *AhOPT7.2* were identified to be Fe-deficiency responsive DEGs. Iron deficiency induced the expression of *AhOPT3.2*/*3.4* but repressed that of *AhYSL3.1*/*3.2* for both cultivars. *AhOPT7.2* expression in Silihong was also induced by Fe deficiency. Cd exposure to Fe-sufficient plants had little influence on *AhOPT* family genes. Only *AhOPT7.3* was identified to be DEG, whose expression was up-regulated by Cd in Fenghua 1. However, Cd exposure and Fe deficiency showed synergy effects on the expression of several *AhOPT* genes. Cd exposure to Fe-deficient plants significantly induced the expression of *AhOPT3.1*/*3.2*/*3.4* and repressed the expression of *AhOPT6.1* for both cultivars. It was also observed that the expression of *AhYSL1.1*/*1.2* and *AhOPT7.1* in the root of Silihong was reduced by Cd exposure and Fe deficiency compared with the control, while that of *AhOPT7.2* was up-regulated.

Nine genes of the *AhOPT* family were identified to be DEGs between Fenghua 1 and Silihong in different treatments (Figure 6B). Under the Fe deficiency condition, Fenghua 1 showed higher expressions of *AhOPT6.1, AhYSL3.1*/*3.2*, and *AhYSL7.4* than Silihong, while the expression of *AhOPT3.4* was higher in Silihong than in Fenghua 1. Under the Cd exposure condition, expressions of *AhOPT6.1, AhOPT7.3*/*7.4, AhYSL6.1*, and *AhYSL7.2* were significantly higher in Fenghua 1 than in Silihong, while *AhYSL7.4* expression was significantly higher in Silihong than in Fenghua 1. Under Cd exposure with Fe deficiency condition, Fenghua 1 showed a higher expression of *AhYSL6.1* but a lower expression of *AhYSL3.2* than Silihong.

To examine relationships between differentially expressed *AhOPT* genes and Cd accumulation, Pearson's correlation analysis was performed. As showed in Figure 6C, the expression of *AhOPT7.1* was significantly and negatively correlated with Cd concentrations in roots (*r* = −0.886, *P* < 0.01) and shoots (*r* = −0.779, *P* < 0.05) as well as the total Cd in plants (*r* = −0.84, *P* < 0.01), but positively correlated with the percentage of Cd in shoots (*r* = 0.743, *P* < 0.05). The percentage of Cd in shoots was also observed to significantly related to the expression of *AhOPT3.1* (*r* = −0.734, *P* < 0.05), *AhOPT3.4* (*r* = −0.728, *P* < 0.05), *AhOPT7.2* (*r* = −0.835, *P* < 0.01), and *AhYSL1.1* (*r* = 0.788, *P* < 0.05). Besides, *AhOPT7.5*/*7.6* and *AhYSL7.7* were not differentially expressed between cultivars or treatments but showed close correlations with Cd accumulation (Supplementary Table 4).

### Prediction and Screening of Transcription Factors of *AhOPT* Genes

**Figure 7:**
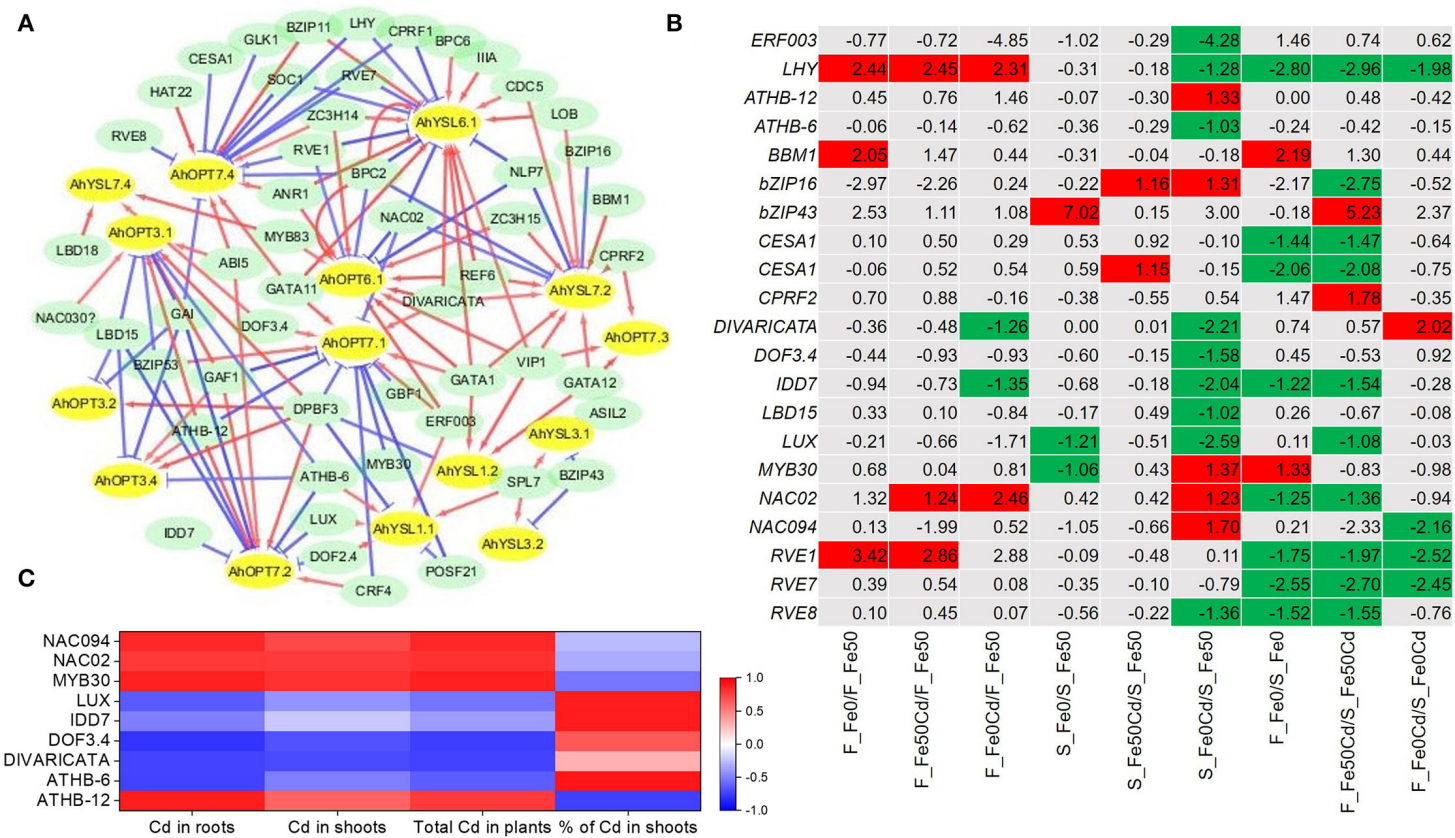
Predicted transcription factors (TFs) of differentially expressed *AhOPT* genes. **(A)** Co-expression network of TFs-*AhOPT*s. The red and blue lines represent positive and negative correlations between TFs and *AhOPT*s, respectively. **(B)** Differentially expressed TFs in Fe-sufficient (Fe50) or Fe-deficient (Fe0) roots of Silihong (S) and Fenghua 1 (F) exposed to 0 or 2 μM CdCl_2_. **(C)** Correlation between the expression of TFs and Cd accumulation.

A total of 69 potential TFs were predicted for the 15 DEGs of the *AhOPT* family. Among them, 57 TFs showed significant correlations with target genes (TGs) (*P* < 0.05). Based on significantly correlated TG-TF pairs, the co-expression network was constructed (Figure 7A). *AhOPT3.1*/*3.2*/*3.4* showed co-expression correlations with *DPBF3* (ABSCISIC ACID-INSENSITIVE 5-like protein 2), *GAI* (DELLA protein GAI), and *LBD15* (LOB domain-containing protein 15). *AhOPT7.1*/*7.2* were significantly correlated with *ATHB-12* (homeobox-leucine zipper protein), *bZIP53* (bZIP transcription factor 53), *CRF4* (ethylene-responsive transcription factor CRF4-like), *DPBF3*, and *GAF1* (zinc finger protein GAI-ASSOCIATED FACTOR 1). *AhYSL3.1*/*3.2* showed significant correlations with *bZIP43* (basic leucine zipper 43) and *SPL7* (squamosa promoter-binding-like protein 7). *AhYSL1.1*/*1.2* shared two co-expressed TFs, such as *SPL7* and *DPBF3*.

Differential gene expression analysis identified 20 DEGs from the 57 co-expressed TFs between treatment or cultivar pairs (Figure 7B). The two cultivars differ from each other in the responses of TF expression to Cd exposure and/or Fe deficiency. Fe deficiency-induced *LHY, BBM1* (AP2-like ethylene-responsive transcription factor), and *RVE1* (protein REVEILLE 1) in Fenghua 1, whereas in Silihong, Fe deficiency up-regulated the expression of *bZIP43* but down-regulated that of *DIVARICATA* and *IDD7* (protein indeterminate-domain 7). Cd-induced *LHY, NAC02* (NAC domain-containing protein 2), and *RVE1* in Fenghua 1, and *bZIP16* [bZIP transcription factor 16-like) and *CESA1* (cellulose synthase A catalytic subunit 1 (UDP-forming)] in Silihong. Cd exposure with Fe deficiency-induced *NAC02* but repressed *LUX* and *MYB30* for both cultivars. The response of TFs to Cd exposure with Fe deficiency was more pronounced in Silihong than in Fenghua 1. Most DEGs of TFs showed higher expression in Silihong than in Fenghua 1.

Pearson's correlation analysis revealed that the expressions of *DIVARICATA, MYB30, NAC02*, and *NAC094* are closely related to Cd concentrations in roots and shoots as well as the total Cd in plants (Figure 7C). The expression of *ATHB-12* and *DOF3.4* is significantly related to root Cd concentrations and the total Cd in plants. A negative correlation was also found between the expression of *ATHB-6* and root Cd concentration. The percentage of Cd in shoots was significantly and positively correlated with the expression of *ATHB-6, IDD7*, and *LUX*, but negatively related to the expression of *ATHB-12* (Figure 7C).

### Verification of the DEG Results

**Figure 8:**
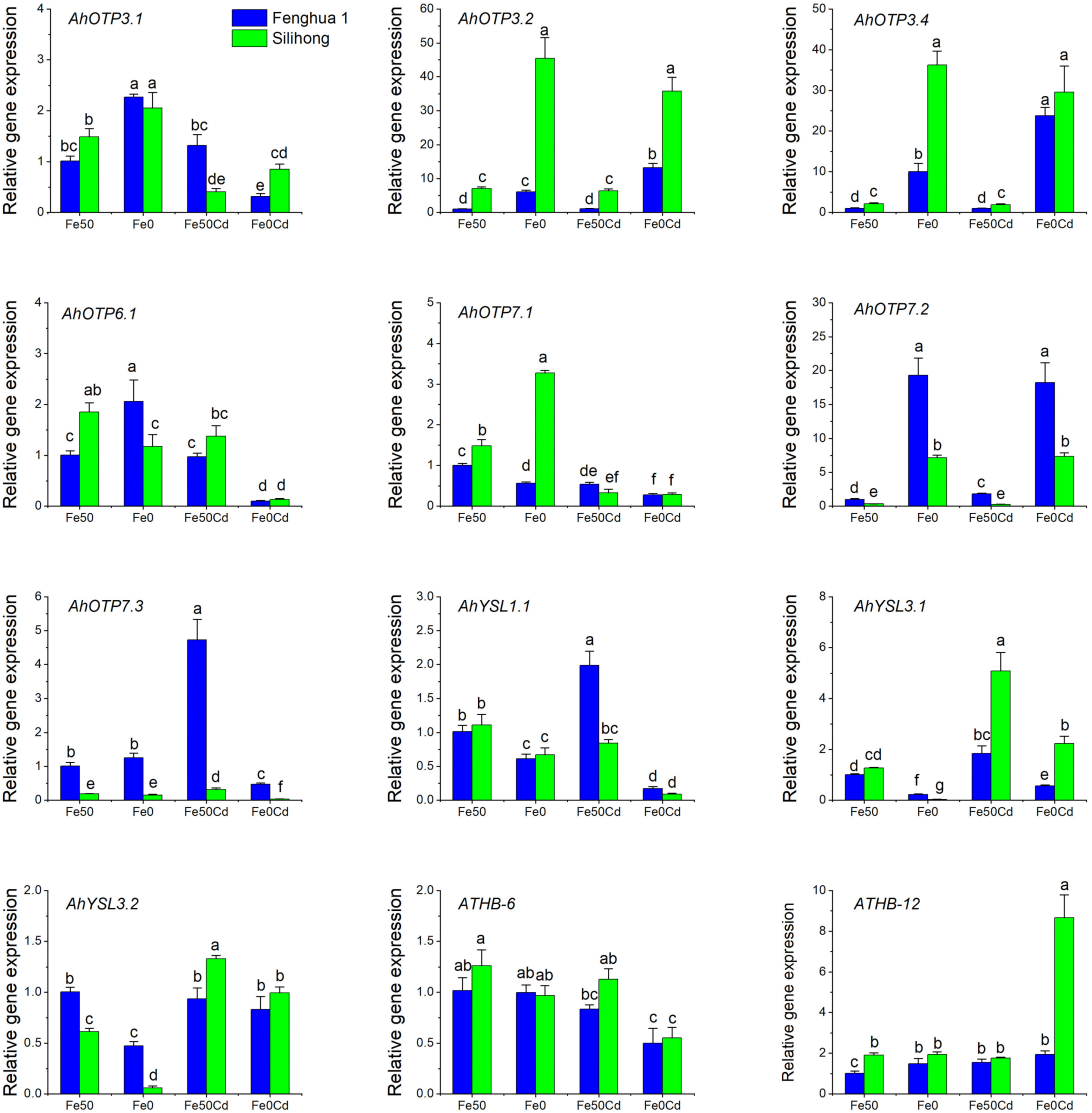
RT-qPCR analysis of ten *AhOPT* genes and two TFs in peanut roots exposed to 0 or 2 μM Cd under Fe-sufficient (Fe50) and Fe-deficient (Fe0) conditions for 14 days. Data (means ± SE, *n* = 3) shared the same letter(s) above the error bars are not significantly different at the 0.05 level by Duncan's multiple range test.

To verify the RNA-seq data, ten DEGs belonging to the OPT family and two TFs were selected for RT-qPCR analysis. As presented in Figure 8, Fe deficiency up-regulated the expression of *AhOPT3.1*/*3.2*/*3.4* and *AhOPT7.2*, but down-regulated *AhYSL1.1* and *AhYSL3.1*/*3.2* for both cultivars. The expression of *AhYSL3.1* was induced by Cd exposure for both cultivars, while that of *AhOPT7.2* was repressed. Cd exposure with Fe deficiency increased the expressions of *AhOPT3.2*/*3.4, AhOPT7.2*, and *ATHB-12*, but decreased those of *AhOPT3.1, AhOPT6.1, AhOPT7.1*/*7.3, AhYSL1.1* and *ATHB-6* (Figure 8). RT-qPCR results showed a good agreement with the RNA-Seq data (Figures 6B, 7B), indicating the reliability of our RNA-Seq data.

## Discussion

Genome-wide identification of the *OPT* family has been performed in several plant species, including poplar (*Populus trichocarpa*) (Cao et al., 2011), grape (*Vitis vinifera*) (Cao et al., 2011), ginseng (*Panax ginseng*) (Su et al., 2019), turnip (*Brassica rapa* var. *rapa*) (Pu et al., 2018), and wheat (Kumar et al., 2019). However, there is little information about the OPT family in peanut. Due to some OPT members have been demonstrated to play crucial roles in the homeostasis of multiple metal ions, including Fe and Cd (Koike et al., 2004; Waters et al., 2006; Stacey et al., 2008; Aoyama et al., 2009; Inoue et al., 2009; Lee et al., 2009, 2012; Chu et al., 2010; Ishimaru et al., 2010; Kakei et al., 2012; Conte et al., 2013; Divol et al., 2013; Mendoza-Cózatl et al., 2014; Zhai et al., 2014; Bashir et al., 2015; Senoura et al., 2017; Zhang et al., 2018b), we speculated that genome-wide identification of *OPT* genes might provide new insights into Fe homeostasis as well as Fe/Cd interaction. Herein, 40 putative *AhOPT* genes were identified in peanut (Table 1). The number of peanut *AhOPT* genes is higher than that of several reported plant species, such as *Arabidopsis* (17) (Koh et al., 2002), rice (27) (Vasconcelos et al., 2008), poplar (20) (Cao et al., 2011), grape (18) (Cao et al., 2011), turnip (28) (Pu et al., 2018), and ginseng (37) (Su et al., 2019). However, it is greatly smaller than that of hexaploid wheat (107) (Kumar et al., 2019). Peanut is an allotetraploid species that essentially possesses two subgenomes (A and B) from ancestral species, *A. duranensis* (AA) and *A. ipaensis* (BB) (Bertioli et al., 2019). Thus, the large number of genes in the *AhOPT* family might be resulted from the WGD during allopolyploidization.

Although AhOPT proteins show a wide variation in TMDs (ranging from 1 to 30), most of them had 11–17 TMDs. The majority of AhOPT proteins shared similar physicochemical properties (Table 1). Overall, most AhOPTs are basic and hydrophobic proteins with high *in vitro* stability over a wide temperature range, which concurred with those obtained from other plant species (Cao et al., 2011; Pu et al., 2018; Su et al., 2019). All AhOPT proteins were predicted to be localized in plasma membranes (Table 1). The results were in accordance with a previous study (Cao et al., 2011; Pu et al., 2018; Su et al., 2019).

Phylogenetic analysis showed that the 40 members of the *AhOPT* family could be divided into two major subfamilies (PT and YS) that was consistent with the previous reports from other species (Koh et al., 2002; Cao et al., 2011; Lubkowitz, 2011; Pu et al., 2018; Su et al., 2019). As expected, peanut shows a closer phylogenetic relationship with *Arabidopsis* in terms of *AhOPT* proteins, compared to rice. The 26 AhOPT proteins assigned to the PT subfamily were further classified into four groups (group 1–4), while the 14 members of the YS subfamily were clustered into three groups (group 6–8) (Figure 1).

Group 1 is composed of two pairs of *AhOPT3* (*AhOPT3.1*/*3.3* and *AhOPT3.2*/*3.4*) derived from WGD. The two gene pairs exhibited considerable differences in the sequence and gene/protein structure. *AhOPT3.1* and *AhOPT3.3* are short sequence genes encoding 132 and 183 aa, with one and three TMDs, respectively, while *AhOPT3.2* and *AhOPT3.4* encode 743 aa with 15 TMDs. Phylogenetic analysis indicates that *AhOPT3* is closely related to *AtOPT3* from *Arabidopsis* and *OsOPT3* from rice. *AtOPT3* is a phloem-specific Fe transporter that is essential for xylem-to-phloem Fe recirculation, shoot-to-root Fe signaling, and redistribution of Fe and Cd in *Arabidopsis* (Stacey et al., 2008; Mendoza-Cózatl et al., 2014; Zhai et al., 2014). In the current study, we found that *AhOPT3.2*/*3.4* are predominantly expressed in leaves and reproductive organs in peanut and the expression of *AhOPT3.1*/*3.2*/*3.4* in roots was induced by Fe deficiency or Fe deficiency with Cd exposure. Similar results have been reported in *Arabidopsis* (Stacey et al., 2006, 2008). The expression of *AhOPT3.1* and *AhOPT3.4* in roots was significantly correlated with the percentage of Cd in shoots. These results indicated that *AhOPT3* might be involved in the transport of Fe and Cd in peanut plants.

Group 2 contained six homologous genes of *AhOPT5*, of which *AhOPT5.1*/*5.2* and *AhOPT5.5*/*5.6* experienced tandem duplication, while *AhOPT5.2*/*5.5* underwent WGD events. Phylogenetic analysis indicates that *AhOPT5* is closely clustered with *AtOPT1/5* from *Arabidopsis* and *OsOPT1/5* from rice. Unfortunately, these genes have not yet been well-functionally characterized. The *Atopt5* mutant line is reported to have higher Pb transport compared with the wild-type (Lubkowitz, 2011). A yeast complementation assay confirmed that OsOPT1 and OsOPT5 could transport Fe(II)–NA and Fe(III)–NA (Vasconcelos et al., 2008). In peanuts, *AhOPT5.1*/*5.6* is highly and preferentially expressed in roots, leaves, and flower tissues, while the remaining four homologous genes showed low expression. The expression of these genes was not affected by Fe deficiency and/or Cd exposure. Therefore, no evidence suggests that the six homologous genes of *AhOPT5* confer the transport of Fe and Cd in peanuts.

Group 3 included four pairs of *AhOPT4* derived from WGD or segmental duplication. Most of them are similar in physicochemical properties and gene/protein structure except *AhOPT4.3*/*4.7*. *AtOPT4* from *Arabidopsis* encodes a broad substrate transporter that can transport a diverse range of tetra- and pentapeptides but not GSH (Koh et al., 2002; Osawa et al., 2006). Yeast harboring *OsOPT4* exhibited rapid growth on a medium containing Fe(II)–NA or Fe(III)–NA (Vasconcelos et al., 2008). In this study, we found that most homologous genes of *AhOPT4* show greatly low expression in all tissues, however, *AhOPT4.4*/*4.8* preferentially transcribed in shoot tips and reproductive tissues. All genes were not affected by Fe deficiency and/or Cd exposure, suggesting that *AhOPT4* is unlikely to be involved in Fe/Cd transport.

Group 4 consists of two homologous genes of *AhOPT6* and six homologous genes of *AhOPT7*. *AhOPT6.1*/*6.2, AhOPT7.1*/*7.5*, and *AhOPT7.3*/*7.6* might be resulted from WGD, while tandem duplication occurred in *AhOPT7.1*/*7.2* and *AhOPT7.4*/*7.5*. All proteins share similar physicochemical properties and structures. RNA-seq data showed that all genes are lowly expressed in peanut tissues except *AhOPT7.2*/*7.4*, which exhibited relatively high expression in shoot tips, leaves, and seed and fruit. Similarly, *OsOPT7* was also reported to expression in root tips, root vascular tissues, shoots, and developing seeds (Bashir et al., 2015). The expression of *OsOPT7* was specifically induced by Fe-deficiency, and *OsOPT7* knockout (*opt7–1*) induces Fe-deficiency responsive genes in plants under Fe-sufficient conditions, indicating that *OsOPT7* may be involved in Fe transport in rice (Bashir et al., 2015). In peanuts, the expression of *AhOPT6.1* and *AhOPT7.1*/*7.2* was altered by Fe deficiency with or without Cd exposure in Silihong, and the expression of *AhOPT7.3* was up-regulated by Cd exposure in Fenghua 1. The expression of *AhOPT7.1* was significantly and negatively correlated with Cd concentrations in roots and shoots as well as the total Cd in plants, but positively correlated with the percentage of Cd in shoots. A significant correlation was observed between the expression of *AhOPT7.2* and percentage of Cd in shoots. Moreover, the expression of *AhOPT7.5* and *AhOPT7.6* also showed close correlations with Cd accumulation. It seems likely that members of group 4 might be involved in Fe/Cd transport in peanut.

Group 6 included eight homologous genes of *AhYSL7*. Among them, *AhYSL7.1*/*7.5* and *AhYSL7.3*/*7.6* underwent WGD, and *AhYSL7.2*/*7.3, AhYSL7.3*/*7.4*, and *AhYSL7.6*/*7.8* experienced tandem duplication. Except for *AhYSL7.1*/*7.5* that were similar in gene/protein structure, the other genes showed significant structural divergences. The majority of genes belonging to group 6 are lowly expressed genes, while *AhYSL7.2 is* preferentially transcribed in shoot tips and reproductive tissues. *OsYSL13* belonging to group 6 is involved in Fe transport in rice plants, particularly from endosperm to embryo in developing seeds (Zhang et al., 2018b). *BjYSL7* encodes a plasma-localized metal–NA transporter that might be involved in the transport of Fe, Cd, and Ni to the shoot and improving heavy metal resistance in plants (Wang et al., 2013). However, all members of group 6 in peanut did not respond to Fe deficiency and/or Cd exposure. Whether these genes are involved in the transport of Fe and/or Cd requires further study.

Group 7 contained *AhYSL6.1*/*6.2* that resulted from WGD. The two genes are similar in gene/protein structure but show different expression patterns. *AhYSL6.2* is highly expressed in all tissues, while *AhYSL6.1* preferentially transcribed in shoot tips and reproductive tissues. The two genes were not affected by Fe deficiency and/or Cd exposure in peanut roots, suggesting that they are unlikely to be involved in the transport of Fe and Cd in peanuts. AtYSL4 and AtYSL6 are located at the internal membranes, such as chloroplast envelope, vacuole membranes and resembling endoplasmic reticulum, and mediating intracellular transport of metal-NA complexes within the cell (Conte et al., 2013; Divol et al., 2013). OsYSL6 is an Mn-NA transporter that is responsible for the detoxification of excess Mn (Sasaki et al., 2011).

Group 8 is composed of *AhYSL3.1*/*3.2* and *AhYSL1.1*/*1.2*. *AhYSL3.1*/*3.2* contains two sets of conserved motifs and domains in protein sequences and two sets of exon/intron in CDS. Thus, we speculated that each of the two genes could be separated into two tandem duplicated genes. The *AhYSL1.1*/*1.2* exhibited little difference similar in physicochemical properties and gene/protein structure. *AhYSL3.1/3.2* showed high expression levels in all tissues, while *AhYSL1.1*/*1.2* was highly and preferentially expressed in roots, leaves, and flower tissues (Figure 4). *AhYSL3.1*/*3.2* were identified to be Fe-deficiency responsive DEGs. The expression of *AhYSL1.1*/*1.2* in the root of Silihong was reduced by Cd exposure and Fe deficiency (Figure 6B). Moreover, a significant correlation was observed between the expression of *AhYSL1.1* and the percentage of Cd in shoots (Figure 6C). These findings indicate that *AhYSL3.1*/*3.2* and *AhYSL1.1*/*1.2* might be essential for Fe transport in peanuts, and *AhYSL1.1* is possibly related to root-to-shoot Cd translocation.

Almost all genes belonging to Group 8 have been functionally characterized in *Arabidopsis* and rice. *AtYSL1, AtYSL2*, and *AtYSL3* are required for the efficient mobilization of Fe, Zn, and Cu from leaves to seeds (Waters et al., 2006; Chu et al., 2010). OsYSL2 is a Fe(II)-NA transporter required for the long-distance transport of Fe(II) -NA and Mn(II)-NA *via* the phloem (Koike et al., 2004; Ishimaru et al., 2010). *OsYSL9* is involved in Fe translocation in plants particularly from endosperm to embryo in developing seeds (Senoura et al., 2017). *OsYSL15* is involved in Fe(III)-DMA uptake from the rhizosphere and in phloem transport of Fe in rice plants (Inoue et al., 2009; Lee et al., 2009). *OsYSL16* is responsible for the allocation of Fe(III)-DMA (Kakei et al., 2012; Lee et al., 2012) and Cu(II)-NA (Zheng et al., 2012; Zhang et al., 2018a) *via* the vascular bundles.

Gene duplication, occurring through polyploidization or unequal crossing over, is a major source of novel genes that contribute to the acquirement of novel functions (Panchy et al., 2016). Expectedly, our results indicate that almost all *AhOPT* genes experienced gene duplication events. Among them, 19 pairs of genes were evolved from WGD, eight pairs from tandem duplication, and one pair from segmental duplication (Figure 3). Homologous genes of *AhOPT4* simultaneously underwent WGD and segmental duplication, and the divergence of segmentally duplicated genes (*AhOPT4.1*/*4.4*) occurred 67.17 Mya ago, which is far earlier than that of WGDs (1.91-7.13 Mya) (Table 2). Homologous genes of *AhOPT5, AhOPT7*, and *AhYSL7* simultaneously underwent WGD and tandem duplication. Tandem duplication events occur either before (i.e., *AhOPT7* and *AhYSL7*) or after WGDs (i.e., *AhOPT5*) (Table 2). Our results indicate that WGD and tandem duplication are the major sources leading to the expansion of the OPT gene family in peanuts. Tandem duplication has been suggested to be a major factor governing the expansion of the OPT gene family in several species (Cao et al., 2011). However, another study revealed that segmental duplication mainly contributes to the expansion of the turnip OPT gene family (Pu et al., 2018).

Before the functional divergence, duplicated genes are usually functionally redundant (Qian et al., 2010). As a result, most duplicated genes are quickly pseudogenized and get lost (Zhang, 2012). To avoid gene loss during evolution processes, the expression of duplicated genes is reduced compared to the ancestral gene (Qian et al., 2010). In the present study, 24 *AhOPT* genes showed low expression levels in all tissues of peanut (cv. Tifrunner) under normal conditions, and 28 genes lowly expressed in the roots of the other two peanut cultivars (cv. Fenghua 1 and cv. Silihong) throughout treatments (Figure 4). All low expression genes are multicopy genes derived from gene duplication. The results concurred with Qian et al. (2010), suggesting that expression reduction might be beneficial for the maintenance of duplicate genes and their functional redundancy.

Duplicated genes, if they survive, would be subject to purifying selection, leading to divergence in both the regulatory and coding regions (Xu et al., 2012). In the current study, eleven pairs of WGD-derived duplicated genes were found to have a shorter divergence time, ranging from 1.74 to 4.06 Mya, and these gene pairs exhibited little divergence in the exon-intron structure (Table 2; Figure 2C). In contrast, the four pairs of duplicated genes (*AhOPT7.1*/*7.4, AhOPT5.1*/*5.4, AhYSL7.2*/*7.7*, and *AhYSL7.3*/*7.6*) with longer divergence time (ranging from 20.74 to 51.56 Mya) showed more considerable structural divergence. The alteration of exon-intron organization concurred with the estimated divergence time from *Ks*, could provide additional evidence to survey the functional divergence of duplicated genes.

Iron deficiency induced the expression of *AhOPT3.2*/*3.4* but repressed that of *AhYSL3.1*/*3.2* for both cultivars (Figure 6B). In *Arabidopsis, YSL3* was demonstrated to translocate metal through vascular parenchyma cells (Waters et al., 2006), while OPT3 is a phloem-specific Fe transporter that is essential for the xylem-to-phloem Fe recirculation (Mendoza-Cózatl et al., 2014; Zhai et al., 2014). Decreased *YSL3* expression during Fe deficiency may decrease long-distance transport of Fe through the xylem and allow more Fe to remain in the vasculature (Waters et al., 2006). Up-regulation of *OPT3* under Fe-limiting conditions would facilitate Fe delivery from xylems to phloems, making it more available to younger tissues (Mendoza-Cózatl et al., 2014; Zhai et al., 2014). It seems likely that the coordination of *AhOPT3.2*/*3.4* and *AhYSL3.1*/*3.2* is responsible for Fe-deficient tolerance in peanuts by altering the Fe transport pathway. As for the two peanut cultivars, Fenghua 1 showed higher expressions of *AhYSL3.1*/*3.2* than Silihong under Fe deficiency condition, while Silihong showed higher expressions of *AhOPT3.4* than Fenghua 1 (Figure 6B). The results indicate that the higher Fe-deficiency tolerance of Silihong might have resulted from the reduced expression of *AhYSL3.1*/*3.2* and increased expression of *AhOPT3.4*.

Cadmium exposure to Fe-sufficient plants had little influence on *AhOPT* family genes. However, Cd exposure and Fe deficiency showed synergy effects on the expression of *AhOPT* genes (Figure 6B). The expression of *AhOPT3.2*/*3.4* was induced by Cd exposure in Fe-deficient plants for both cultivars, while *AhOPT6.1* was repressed. It was also observed that the expression of *AhYSL1.1*/*1.2* and *AhOPT7.1* in the root of Silihong was reduced by Cd exposure and Fe deficiency, while that of *AhOPT7.2* was up-regulated. The expression of *AhOPT7.1* was negatively correlated with Cd concentrations in roots and shoots as well as the total Cd in plants but positively correlated with the percentage of Cd in shoots (Figure 6C). The percentage of Cd in shoots was also observed to significantly related to the expression of *AhOPT3.1, AhOPT3.4, AhOPT7.2*, and *AhYSL1.1*. As mentioned above, *AtOPT3* has been shown to be a phloem-specific Fe transporter involved in the redistribution of Cd in *Arabidopsis* (Stacey et al., 2008; Mendoza-Cózatl et al., 2014; Zhai et al., 2014). *OsOPT7* is a plasma membrane-localized Fe transporter expressed in all tissues near root tips in Fe-deficient roots, particularly in the epidermis and vascular tissues (Bashir et al., 2015). Hence, our findings suggest that *AhOPT3.1, AhOPT3.4, AhOPT7.1, AhOPT7.2*, and *AhYSL1.1* might be involved in the effects of Fe deficiency on Cd accumulation and translocation in peanut plants.

In agreement with previous studies (Liu et al., 2017; Tian et al., 2019), we found that Silihong showed a higher capacity for uptake and translocation of Cd from roots to shoots than Fenghua 1. Under Fe-sufficient conditions, Fenghua 1 showed a higher expression of *AhOPT7.3* and *AhOPT6.1* than Silihong (Figure 6B). The expression of *AhOPT7.3* was up-regulated by Cd in Fenghua 1, while *AhOPT6.1* was repressed by Cd for both cultivars under Fe-deficient conditions. It seems likely that increased *AhOPT7.3* expression may enhance Cd tolerance by reducing Cd uptake in Fenghua 1. Likewise, reduced *AhOPT6.1* expression in the root of Fe-deficient plants increases Cd uptake, which is more pronounced in Silihong. Although the expression of *AhYSL3.2* in Silihong was higher than that in Fenghua under Cd exposure with Fe-deficiency conditions, it was unaffected by Cd regardless of Fe supply. It seems unlikely that *AhYSL3.2* was involved in the cultivar difference in Cd uptake and accumulation in peanuts. Taken together, higher expression of *AhOPT7.3* and *AhOPT6.1* might be responsible for low Cd accumulation in Fenghua 1.

MicroRNAs are generally believed to down-regulate the expression of target genes by cleaving mRNA or inhibiting the translation of target genes (Bartel, 2009). *MiR408* has been proposed to regulate copper homeostasis by down-regulating the copper-containing proteins (laccase and plantacyanin) in *Arabidopsis* (Abdel-Ghany and Pilon, 2008). *MiR156a* and *miR156c* play dominant roles in regulating abiotic stress resistance through a *miR156*-*SPL* regulatory pathway (Cui et al., 2014; Wang et al., 2021). The *miR167* has been reported to target the mRNAs encoding the ARF6, ARF8, and IAR3, regulating auxin signaling and homeostasis in *Arabidopsis* (Wu et al., 2006; Kinoshita et al., 2012; Yao et al., 2019). Another study demonstrated that *BnNRAMP1b* is a target of *miR167* in *Brassica napus* (Meng et al., 2017). The expression of *miR167* and *miR156* was down-regulated by Ca deficiency in peanut embryos (Yu et al., 2019). Similar results were reported in the roots and shoots of the high-Fe rice line under Fe deficiency (Agarwal et al., 2015). In peanut, *AhOPT3.2/3.4* were predicted to be targets of *ahy-miR156a* and *ahy-miR156c*, and *AhYSL3.1*/*3.2, AhYSL7.4*/*7.7*, and *AhYSL7.2*/*7.8* might be the potential targets of *ahy-miR159, ahy-miR167-3p*, and *ahy-miR408-5p*, respectively (Table 3). Because most of the target genes identified in the *AhOPT* family possibly participate in the transport of metal ions, including Fe and Cd, these miRNA might play a role in metal transport by post-transcriptionally repressing *AhOPT* genes.

Transcription factors are recognized as the key regulators of gene expression. In this study, a total of twelve TFs were identified for the five Fe-deficiency responsive DEGs (*AhOPT3.1*/*3.2*/*3.4* and *AhYSL3.1*/*3.2*), including *ABI5, ASIL2, ATHB-12, ATHB-6, bZIP43, bZIP53, DPBF3, GAF1, GAI, LBD15, NAC030*, and *SPL7*. Among them, *DPBF3, GAI*, and *LBD15* were predicted as common TFs for *AhOPT3.1*/*3.2*/*3.4*, and *bZIP43* and *SPL7* for *AhYSL3.1*/*3.2*. Besides, *ATHB-12, ATHB-6*, and *GAF1* are common TFs of *AhOPT3.1*/*3.4*. Moreover, *ATHB-12, ATHB-6, bZIP43*, and *LBD15* were affected by Fe-deficiency with or without Cd in Silihong (Figure 7B). These findings suggested that *ATHB-12, ATHB-6, bZIP43*, and *LBD15* might be involved in Fe-deficiency responses in peanuts by regulating the corresponding Fe-deficiency responsive targets.

Pearson's correlation analysis revealed that *DIVARICATA, MYB30, NAC02, ATHB-12*, and *DOF3.4 were* closely related to Cd uptake and accumulation in plants, while *ATHB-12, ATHB-6, IDD7*, and *LUX* were significantly correlated with root-to-shoot Cd translocation (Figure 7C). Interestingly, *ATHB-12, DIVARICATA, NAC02, MYB30*, and *DOF3.4* were predicted as potential TFs of *AhOPT7.1, ATHB-12, ATHB-6, IDD7*, and *LUX* as potential TFs of *AhOPT7.2, ATHB-12* and *ATHB-6* as potential TFs of *AhOPT3.1* and *AhOPT3.4*, and *ATHB-6* and *LUX* as potential TFs of *AhYSL1.1* (Figure 7A). All these target genes showed significant correlations with the transport of Cd in peanut plants. Therefore, *ATHB-12, ATHB-6, DIVARICATA, MYB30, NAC02, DOF3.4, IDD7*, and *LUX* might be involved in Fe/Cd interactions by regulating *AhOPT* genes in peanut plants.

## Conclusion

**Figure 9:**
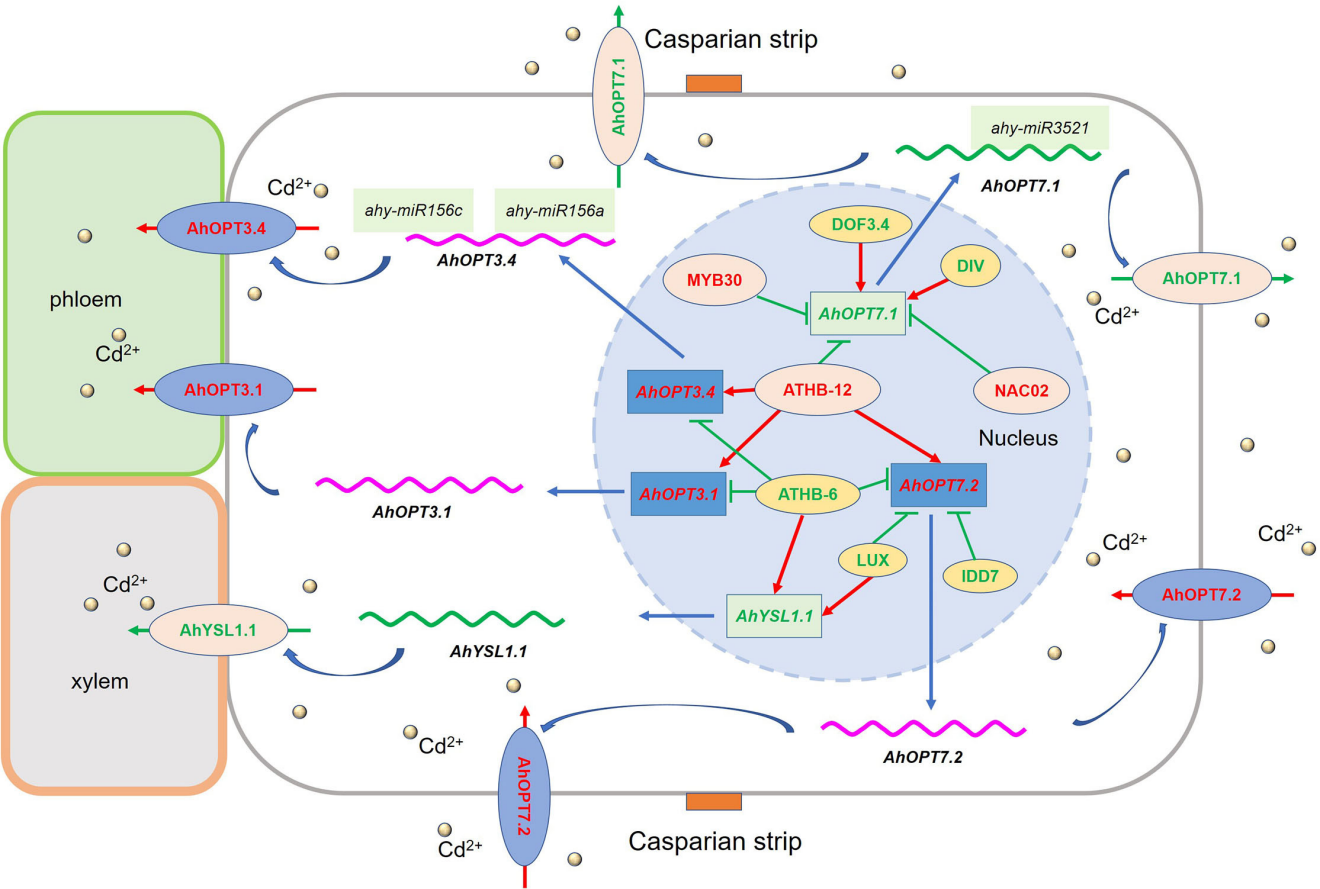
The putative model explains the regulation of Cd acquisition and translocation in the peanut roots under iron deficiency. Gene names highlighted with red or green color indicate that they were upregulated and downregulated by Cd exposure with iron deficiency, respectively. The red and blue lines in the nucleus represent positive and negative correlations between TFs and *AhOPT*s, respectively. The localization of these genes was based on the data previously published in other plant species, such as *Arabidopsis* and rice.

A total of 40 *AhOPT* genes were identified in peanuts, which was divided into two subfamilies (PT and YS). Most *AhOPT* genes underwent gene duplication events particularly WGD and tandem duplication. Clustered members generally have similar gene and protein structures. However, structural divergences occurred in the majority of the duplicated gene pairs. Most *AhOPT* genes showed reduced expression under normal conditions, which may be beneficial for the maintenance of duplicate genes and their functional redundancy. Transcription analysis revealed that *AhOPT3*/*6*/*7* and *AhYSL1*/*3* might be involved in the transport of Fe and/or Cd in peanut plants. *AhOPT3.2*/*3.4* and *AhYSL3.1*/*3.2* might be responsible for Fe deficiency tolerance, and *AhOPT3.1*/*3.4, AhOPT7.1*/*7.2*, and *AhYSL1.1* might be involved in Fe/Cd interaction (Figure 9). These genes might be regulated by TFs, including *ATHB-12, ATHB-6, DIVARICATA, MYB30, NAC02, DOF3.4, IDD7*, and *LUX* (Figure 9). Reduced expressions of *AhYSL3.1*/*3.2* and higher *AhOPT3.4* expression might contribute to higher Fe-deficiency tolerance in Silihong, and higher expression of *AhOPT7.3* and *AhOPT6.1* might be responsible for low Cd accumulation in Fenghua 1. The results confirmed the role of *AhOPT* genes in the transport of Fe and Cd in peanuts and provided new clues to understanding mechanisms underlying Fe/Cd interactions.

## Data Availability Statement

The original contributions presented in the study are included in the article/Supplementary Material, further inquiries can be directed to the corresponding author/s.

## Author Contributions

CW, XW, JL, JG, and ZT carried out most of the experimental work with assistance from GS and ZZ. GS and ZZ were responsible for the experimental design. GS, CW, and XW carried out data analyses. GS and CW wrote and revised the manuscript. All authors contributed to the article and approved the submitted version.

## Funding

This work was supported by grants from the Natural Science Foundation of Anhui Province (Grant Number 2108085MC83), the Natural Science Foundation for Colleges and Universities of Anhui Province (Grant Numbers KJ2020ZD83 and KJ2019A0587), and the Innovation Foundation for Graduate Students of Huaibei Normal University (Grant Number YX2021025).

## Conflict of Interest

The authors declare that the research was conducted in the absence of any commercial or financial relationships that could be construed as a potential conflict of interest.

## Publisher's Note

All claims expressed in this article are solely those of the authors and do not necessarily represent those of their affiliated organizations, or those of the publisher, the editors and the reviewers. Any product that may be evaluated in this article, or claim that may be made by its manufacturer, is not guaranteed or endorsed by the publisher.

## Footnotes

^1^ https://phytozome-next.jgi.doe.gov

^2^ https://www.ebi.ac.uk/Tools/hmmer/search/hmmscan

^3^ https://itol.embl.de/itol.cgi

^4^ https://web.expasy.org/protparam

^5^ http://topcons.net/

^6^ http://www.csbio.sjtu.edu.cn/bioinf/plant-multi/

^7^ https://meme-suite.org/meme/tools/meme

^8^ http://pfam.xfam.org/search#tabview=tab1

^9^ http://gsds.gao-lab.org/

^10^ https://www.peanutbase.org/gene_expression/atlas

^11^ http://plantregmap.gao-lab.org/network.php

^12^ https://www.peanutbase.org/

## Abbreviations

CDS: Coding sequence
Chr: Chromosomes
GRAVY: grand average of hydropathicity
FPKM: Fragments Per Kilobase of exon model per Million mapped reads
*K*a: The number of nonsynonymous substitutions per nonsynonymous site
*K*s: The number of synonymous substitutions per synonymous site
OPT: Oligopeptide transporters
pI: isoelectric point
RT-qPCR: Real-time quantitative PCR
TMDs: Transmembrane domains.
